# Network Physiology of Cortico–Muscular Interactions

**DOI:** 10.3389/fphys.2020.558070

**Published:** 2020-11-26

**Authors:** Rossella Rizzo, Xiyun Zhang, Jilin W. J. L. Wang, Fabrizio Lombardi, Plamen Ch. Ivanov

**Affiliations:** ^1^Keck Laboratory for Network Physiology, Department of Physics, Boston University, Boston, MA, United States; ^2^Evolutionary Systems Group Laboratory, Department of Physics, University of Calabria, Rende, Italy; ^3^Department of Physics, Jinan University, Guangzhou, China; ^4^Institute of Science and Technology Austria, Klosterneuburg, Austria; ^5^Division of Sleep Medicine, Harvard Medical School, Brigham and Women's Hospital, Boston, MA, United States; ^6^Institute of Solid State Physics, Bulgarian Academy of Sciences, Sofia, Bulgaria

**Keywords:** network physiology, dynamic networks, time delay stability, bursts, synchronization, brain waves, muscle tone, sleep

## Abstract

Skeletal muscle activity is continuously modulated across physiologic states to provide coordination, flexibility and responsiveness to body tasks and external inputs. Despite the central role the muscular system plays in facilitating vital body functions, the network of brain-muscle interactions required to control hundreds of muscles and synchronize their activation in relation to distinct physiologic states has not been investigated. Recent approaches have focused on general associations between individual brain rhythms and muscle activation during movement tasks. However, the specific forms of coupling, the functional network of cortico-muscular coordination, and how network structure and dynamics are modulated by autonomic regulation across physiologic states remains unknown. To identify and quantify the cortico-muscular interaction network and uncover basic features of neuro-autonomic control of muscle function, we investigate the coupling between synchronous bursts in cortical rhythms and peripheral muscle activation during sleep and wake. Utilizing the concept of time delay stability and a novel network physiology approach, we find that the brain-muscle network exhibits complex dynamic patterns of communication involving multiple brain rhythms across cortical locations and different electromyographic frequency bands. Moreover, our results show that during each physiologic state the cortico-muscular network is characterized by a specific profile of network links strength, where particular brain rhythms play role of main mediators of interaction and control. Further, we discover a hierarchical reorganization in network structure across physiologic states, with high connectivity and network link strength during wake, intermediate during REM and light sleep, and low during deep sleep, a sleep-stage stratification that demonstrates a unique association between physiologic states and cortico-muscular network structure. The reported empirical observations are consistent across individual subjects, indicating universal behavior in network structure and dynamics, and high sensitivity of cortico-muscular control to changes in autonomic regulation, even at low levels of physical activity and muscle tone during sleep. Our findings demonstrate previously unrecognized basic principles of brain-muscle network communication and control, and provide new perspectives on the regulatory mechanisms of brain dynamics and locomotor activation, with potential clinical implications for neurodegenerative, movement and sleep disorders, and for developing efficient treatment strategies.

## 1. Introduction

The human body is composed of diverse organ systems, each with its own regulatory mechanisms and complex dynamical behavior. Organ systems continuously interact and coordinate their dynamics to ensure vital functions, to allow the body to perform daily activities, and facilitate restoring functions during night sleep. Organ-to-organ interactions occur at multiple levels and spatio-temporal scales to produce distinct physiologic states, e.g., wake and sleep. Mapping the network of organ interactions is thus of primary importance to fully understand basic physiologic states and functions, rigorously discriminate between healthy and pathological behaviors, and understand complex diseases associated with alterations and breakdown of networked interactions across levels in the human organism. A new field, Network Physiology, has been established to address the fundamental question of how distinct physiologic states and functions emerge out of organ network interactions (Bashan et al., 2012; Ivanov and Bartsch, 2014; Ivanov et al., 2016, 2017). Novel methodologies and approaches have been recently developed within the framework of Network Physiology to investigate brain-organ and organ-organ interactions and their association to different physiologic states (Faes et al., 2014, 2015; Bartsch et al., 2015; Liu et al., 2015b; Porta and Faes, 2015; Lin et al., 2016; Moorman et al., 2016).

In this context, the functional network involved in the neural control of the muscular system remains poorly understood. In particular, how different brain rhythms communicate and control diverse muscle groups, and how different frequency components of muscle tone activation respond to signals from the brain is to a large extent not known. The muscular system comprises hundreds of muscles of different types attached to the skeletal system, and is responsible for body stability, movement and control. Skeletal muscles are made of a collection of muscle fibers, which are broadly classified as fast and slow based on their speed of shortening (Scott et al., 2001). Muscles vary considerably in size, shape, and arrangement of fibers. Their common, predominant characteristic is contractibility, and nearly all movements in the body result from muscle contraction. Necessary inputs to achieve both simple and articulated body movements are coordinated by certain brain areas and transmitted to the different muscles by the motor neurons in the spinal cord, where distinct sets of locomotor modules control locomotion (Yokoyama et al., 2016; Rendeiro and Rhodes, 2018; Zandvoort et al., 2019).

It has long been known that movements induce frequency specific changes in the electroencephalography (EEG) (Jasper and Penfield, 1949; Chatrian et al., 1958). Changes in the spectral power in the α (8-14 Hz) and β (15-30 Hz) frequency bands can be observed during both voluntary and passive movements (Pfurtscheller and Aranibar, 1977; Leocani et al., 1997; Pfurtscheller and da Silva, 1999; Cheyne, 2013). Moreover, movement-related cortical activity in the γ band (30–100 Hz) has been demonstrated in both magnetoencephalography (MEG) (Tecchio et al., 2008) and scalp EEG recordings (Ball et al., 2008; Darvas et al., 2010), and increased γ activity in the electrocorticogram (ECoG) in awake patients performing sustained muscle contractions has also been reported (Crone et al., 1998). Human cortical 40 Hz rhythms were related to electromyographic (EMG) rhythmicity (Salenius et al., 1996), and cortical control of human motor neuron firing was associated with isometric muscle contractions (Salenius et al., 1997). Furthermore, studies on muscular coordination between the limbs provided applications for neurological rehabilitation after neurotrauma (Zehr et al., 2016). Recent works have focused on the synchronization between rhythmical activity in the motor cortex and muscular activity employing cortico-muscular coherence (CMC). CMC is usually observed during periods of muscular contraction, and has been reported in a number of studies involving both EEG and MEG (Conway et al., 1995; Boonstra et al., 2009; Cheyne, 2013). Specifically, it has been shown that oscillations in the motor cortex, particularly in the β frequency band, can exhibit coherence with peripheral EMG activity during sustained motor contractions, which suggests a possible role of cortical rhythms in direct cortico-spinal drive to the muscle (Conway et al., 1995; Brown et al., 1998; Baker et al., 1999). CMC has been also observed at higher γ band frequencies during dynamic movements (Omlor et al., 2007) or during sustained isometric contractions (Brown et al., 1998). Moreover, studies of neuromotor control showed that task-specific combinations of muscle activity are represented in the cortex, and that these representations are involved in balance control and short-term balance training (Zandvoort et al., 2019).

Current approaches to cortico-muscular coordination focus on associations and synchronous activation between individual brain rhythms at specific cortical areas (e.g., motor cortex, hyppocampus), and peripheral muscle activity during specific movement tasks or exercises (walking, running, etc.) (van Wijk et al., 2012; Rendeiro and Rhodes, 2018). EMG signal decomposition techniques have also been employed to better understand the muscle activation of different muscle groups during different locomotor modes (Yokoyama et al., 2016). However, the muscular system constantly supports the body across different vigilant or physiologic states, which are characterized by the coordinated synchronous activation of different muscle groups that is specific for each movement (Kerkman et al., 2018; Boonstra et al., 2019), as well as by an intense cross-talk among brain rhythms within and across different cortical areas (Bashan et al., 2012; Bartsch et al., 2015; Liu et al., 2015a; Wang et al., 2019; Lin et al., 2020; Lombardi et al., 2020a,b). Default control and coordination of hundreds of skeletal muscles in relation to distinct physiologic states must require both the collective behavior of large population of neurons, i.e., brain rhythms and brain rhythms cross-talk, and a communication network of functional interactions between cortical rhythms and muscular system. Such a network of interactions should provide the necessary brain-muscle coordination in the absence of specific body tasks or targeting movements during rest and sleep—e.g., for postural adjustments, adaptability, sensory feedback—as well as the adequate responsiveness in each vigilant state. Despite the fundamental role played by this network of communication in our basic daily activities and its relevance in achieving efficient motor rehabilitation strategies, the relationship between cortical rhythms and default muscles activity is largely not known. In particular, little is known on the dynamical cross-talk between brain rhythms across cortical areas and differentiated muscle tone rhythms, and on how these frequency-based communications integrate as a dynamic network of cortico-muscular interactions across physiologic states. Recent empirical investigations demonstrated that brain waves interactions are characterized by distinct coupling profiles and network plasticity that are essential to generate physiological states and functions (Liu et al., 2015a; Lin et al., 2020). Thus, we hypothesize that network interactions of brain waves and muscle activity may also reflect changes in physiologic regulation as a function of physiologic states. In these functional cortico-muscular networks we represent muscle activation through different frequency domains corresponding to the role and frequency of activation of slow and fast muscle fibers in a given muscle group (Garcia-Retortillo et al., 2020). Further, we ask how cortico-muscular networks hierarchically reorganize with transitions across physiologic states, e.g., wake and sleep, sleep stages.

We investigate the coupling between physiologically relevant brain rhythms at different cortical locations with peripheral EMG activity across four major, well-defined physiologic states—Wake, REM, Light Sleep (LS), Deep Sleep (DS). We aim to map the default brain-muscle interaction network corresponding to low level of physical activity and absence of directed and targeting movements during sleep and during quiet restful wake, and to uncover basic features of autonomic regulation of muscle activation. This communication network comprises the ensemble of frequency-specific pathways involved in the synchronous dynamics of the EEG and EMG signals. We study the brain-muscle cross-talk over long-term recordings during night-time sleep, when the muscle activation is low due to absence of conscious movements. Therefore, under these conditions changes in coupling dynamics reflect underlying mechanism of physiologic regulation specific for different physiologic states and are modulated by transition from one physiological state to another. By dissecting dynamical changes in the structure and topology of the brain-muscle interaction network across physiologic states, our study aims to establish the basic features of autonomic regulation of muscle activation. We hypothesize that, because of the different types of muscle fibers and the variety of fibers arrangements observed in the muscular systems (Scott et al., 2001): (i) the brain-muscle communication takes place over interaction channels corresponding to a range of physiologically relevant EEG and EMG frequency bands; and (ii) the strength of the interactions across these channels is modulated in relation to the transition from one physiologic state to another.

To uncover principles of control and basic functional pathways in the default communication network between brain and peripheral muscles, we focus on brain and muscle activity during night sleep, when influences of physical activity are minimal, and muscle tone activation is reduced. To this end, we utilize a Network Physiology framework (Ivanov and Bartsch, 2014; Ivanov et al., 2016, 2017) and a recently developed method based on the concept of Time Delay Stability (TDS) (Bashan et al., 2012). This approach is inspired by observations of coordinated bursting activity in the output dynamics of physiological systems, and infers coupling based on the stability of the time delay with which bursts of activation in the output dynamics of a given system are followed by corresponding bursts in the signal output of other systems. The TDS method is robust and can track changes in the intensity of interaction among organ systems with transitions across physiologic states (Bartsch and Ivanov, 2014; Bartsch et al., 2015; Liu et al., 2015b; Lin et al., 2016). This method provides a general framework—not limited to the analysis of bursting signals—that can be applied to diverse systems with very different types of output dynamics (oscillatory, stochastic or mixed), and does not have the limitations of synchronization methods applicable only to systems with oscillatory dynamics.

By probing the coupling through the time delay in the bursting dynamics in the brain represented by physiological relevant cortical rhythms and peripheral muscle output signals, we establish the first detailed brain-muscles interaction networks characterizing basic physiologic states, and we show that the default brain-muscle network comprises state-specific patterns of communication involving several frequency bands—not only beta or gamma as shown by CMC during motor contraction. Crucially, we discover key interaction profiles characterizing cortico-muscular communication under autonomic regulation even at low level of physical activity during rest and sleep, and we identify the main frequency bands through which the default brain-muscle communications are mediated during each physiologic state. Importantly, we find that cortico-muscular interaction profiles and the related networks change with transition from one physiologic state to another (sleep vs. wake, and different sleep stages), and thus, are a unique signature of physiologic state and function, allowing to discriminate different physiologic and pathologic conditions.

## 2. Materials and Methods

### 2.1. Data

We analyze high-frequency output signals obtained from 36 healthy young subjects (ages between 20 and 40, average 29 years), synchronously and continuously recorded during night-time sleep (average record duration 7.8 h). Data were divided in 30 s epochs and scored as Wake, REM, LS, and DS. Sleep stage scoring was performed based on standard criteria (Bartsch et al., 2015; Liu et al., 2015b). Analyzed data include EEG (sampling rate 100 Hz for two subjects, 200 Hz for 15 subjects, and 256 Hz for 19 subjects) from six scalp locations (frontal left-Fp1, frontal right-Fp2, central left-C3, central right-C4, occipital left-O1, and occipital right-O2; reference electrodes are M1 for the right hemisphere and M2 for the left hemisphere) and the EMG (sampling rate 200 Hz for 17 subjects and 256 Hz for 19 subjects) of chin muscle and left leg muscle. Before the mounting of the EMG electrodes, the participants' skin is shaved and cleaned using alcohol and left to dry for the 60 s to reduce the myoelectrical impedance. The following muscles are investigated simultaneously during night sleep: the anterior tibialis (leg) and the mentalis (chin). The electrodes for the anterior tibialis (pre-gelled Ag/AgCl bipolar surface electrodes) are placed at 1/3 on the line between the tip of the fibula and the tip of the medial malleolus with an interelectrode distance of 20 mm. The orientation of the electrodes corresponds to the direction of the line between the tip of the fibula and the tip of the malleolus. The reference electrode is located in the ankle. For the mentalis muscle, 8-mm-diameter surface pre-gelled electrodes are placed on the mentalis equidistant to the median line with an inter-electrode distance of 10 mm. The ear lobe is used as a reference point. After the electrodes are secured, a quality check is performed to ensure EMG signal validity. Data used in this study are multi-channel physiologic recordings from EU SIESTA databases (Klösch et al., 2001). All participants provided written informed consent. The research protocol was approved (protocol number 3380X) by the Institutional Review Boards of Boston University (Boston, MA, USA) and was conducted according to the principles expressed in the Declaration of Helsinki.

### 2.2. Signal Pre-processing

Data were visually inspected to remove noisy segments. Such segments were usually located at the beginning and the end of the recordings, and are related to the procedure of electrode placement/removal, or to electrode misplacement. Power-line interferences were removed using a 50 Hz notch filter designed in Matlab (Mathworks), and signals were bandpass filtered in the range (0.5–98.5) Hz. To compare EEG and EMG signals and study their physiological interaction: the spectral power of seven frequency bands of both the EEG and the EMG was parallelly extracted in moving windows of 2 s with a 1 s overlap: δ (0.5–3.5 Hz), θ (4–7.5 Hz), α (8–11.5 Hz), σ (12–15.5 Hz), β (16–19.5 Hz), γ_1_ (20–33.5 Hz), and γ_2_ (34–98.5 Hz). This defines a time series *S*^ν^—with ν = 1, ..., *N*, and *N* number of windows—for each frequency band, with a temporal resolution of 1 s. The spectral power *S*(*f*) has been calculated as $S\left(f\right)=|F\left(f\right){|}^{2}/\left(W\cdot F_{s}\right)$, where *F*(*f*) is the Fourier transform, *W* is the window size, and *F*_*s*_ is the sampling frequency. The Fourier transform has been evaluated using the fast Fourier transform (FFT) algorithm in Matlab. The spectral power in a given window ν and in a given frequency band Δ*f* is defined as

$$S^{\nu }\left(\Delta f\right)=\int_{f_{1}}^{f_{2}}S^{\nu }\left(f\right)df$$

where *f*_1_ and *f*_2_ are the lower and upper bound of the band.

### 2.3. Time Delay Stability (TDS) Method

**Figure 1:**
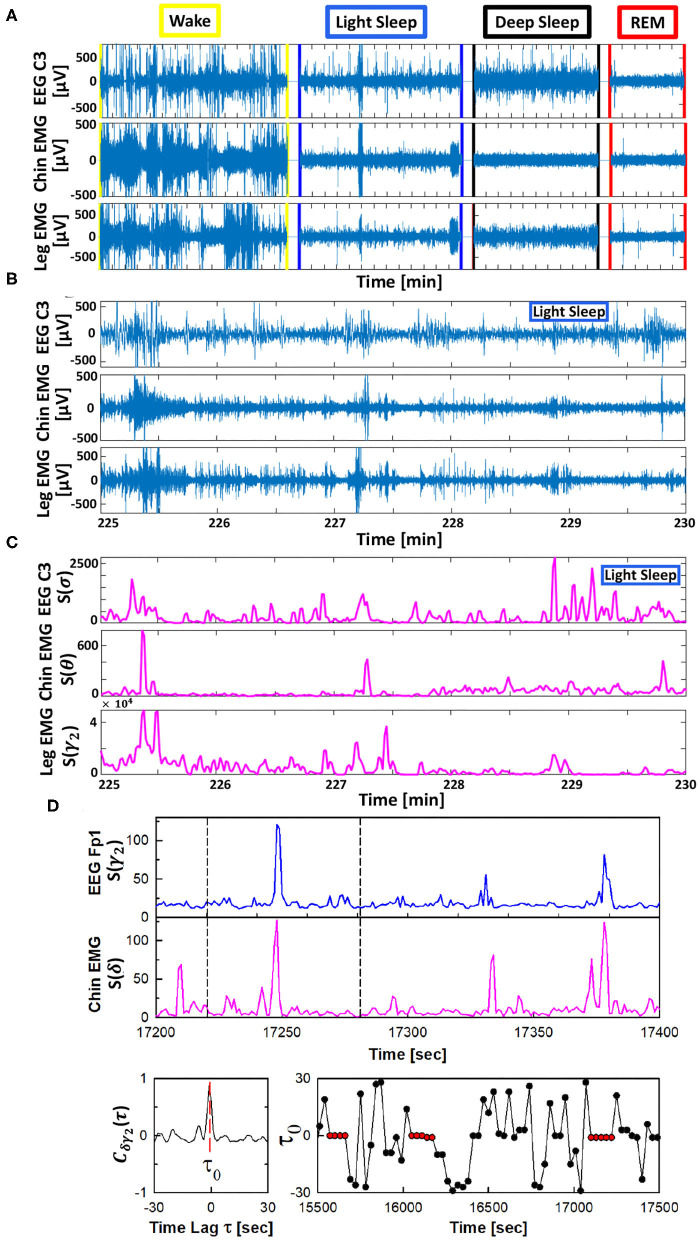
Schematic presentation of the Time Delay Stability (TDS) method. Based on the concept of time delay stability the TDS method is designed to quantify coupling in diverse physiological systems with bursting dynamics. **(A)** Segments of 10-15 min raw signals from brain central EEG C3, chin muscle tone EMG and leg muscle tone EMG channels during different physiological states. **(B)** Magnified section (5 min segments) of the raw signal with bursting morphology for brain C3 and chin and leg muscle tone during LS. **(C)** Spectral power *S*(*f*) for a combination of chosen frequency bands for the raw data shown in **(B)**. **(D)** (top panels) Brain EEG spectral power *S*(*f*) in the γ_2_-band (from frontal Fp1 channel) and chin muscle tone EMG spectral power in the δ-band. Segments with synchronous bursts in *S*(γ_2_) and *S*(δ) lead to a pronounced peak in the cross-correlation *C*(τ) at time lag τ_0_ (shown in the left bottom panel for the 60 s window marked by vertical dashed lines in the top panels). Periods with stable time delay are characterized by constant τ_0_ (red dots in **B**, right panel). Cross-correlation *C*(τ) is performed for overlapping windows of 60 s with a moving step of 30 s, and the time lag τ_0_ corresponding to the peak of *C*(τ) in each window is recorded (shown in the right bottom panel), where consecutive red dots indicate periods of time delay stability with constant τ_0_ (see Materials and Methods section 2.3). Long periods of constant time delay τ_0_ indicate strong TDS coupling, represented by strong links in the network of physiologic interactions between cortical EEG rhythms and muscle tone frequency bands. The TDS approach is general, and can identify and quantify interactions between diverse systems with different dynamic characteristics across physiological states.

The TDS method is a novel approach specifically developed to identify and quantify pair-wise coupling and network interactions of diverse dynamical systems (Bashan et al., 2012). This approach is inspired by observations of coordinated bursting activity in the output dynamics of diverse systems (Figure 1).

**Figure 2:**
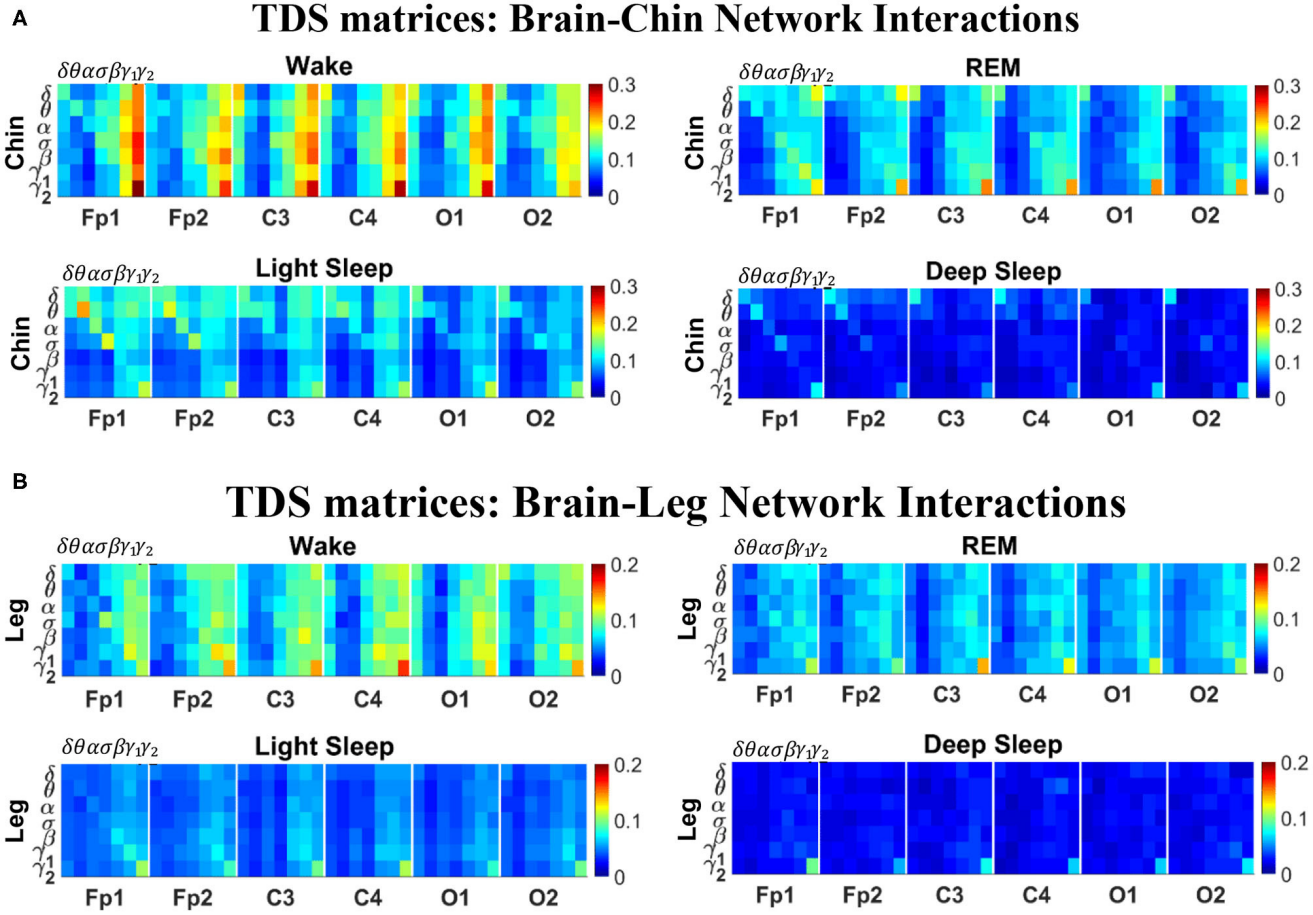
TDS matrix representation of brain-muscle network connectivity across physiologic states. Group-averaged Time Delay Stability (TDS) matrices represent physiological interactions during wake, REM, light and deep sleep. Matrix elements show the coupling strength between seven physiologically-relevant cortical rhythms (δ, θ, α, σ, β, γ_1_, γ_2_) derived from six EEG channels (x-axis: Frontal Fp1 and Fp2; Central C3 and C4; Occipital O1 and O2) and the corresponding EMG frequency bands (y-axis) representing chin and leg muscle activation (shown in **A,B**). Coupling (network links) strength is quantified by the fraction of time (out of the total duration of a given sleep stage throughout the night) when TDS is observed. Matrix elements are obtained by quantifying the TDS for each pair of EEG vs. EMG bands after calculating the weighted average of all subjects in the group (Methods section 2.3). Color code indicates TDS coupling strength. The average number of synchronized bursts per minute corresponding to periods of time delay stability depends on the physiologic state: 0.21 ± 0.08 for Wake, 0.17 ± 0.07 during REM, 0.15 ± 0.05 during LS and 0.08 ± 0.04 during DS. Brain-chin and brain-leg network interactions exhibit pronounced sleep-stage stratification: strong coupling across all pairs of EEG vs. EMG bands during wake, intermediate for REM and light sleep, and weak coupling during deep sleep. Notably, high frequency cortical rhythms are the dominant mediator of both brain-chin and brain-leg interactions (warmer colors for vertical columns representing coupling of β, γ_1_, γ_2_ brain waves with EMG muscle bands)—characteristic that is consistently observed across all sleep stages.

The TDS method is based on the concept of time delay stability. Integrated physiologic systems are coupled by non-linear feedback and/or feed forward loops with a broad range of time delays. Thus, bursting activities in one system are always followed by bursts in signals from other coupled systems. TDS quantifies the stability of the time delay with which bursts in the output dynamics of a given system are consistently followed by corresponding bursts in the signal output of other systems (Figure 1)—periods with a constant time delay between bursts in two systems indicate stable interactions. Correspondingly stronger coupling between systems results in longer periods of TDS (Figure 2). Thus, the links strength in the physiologic networks we investigate is determined by the percentage of the time when TDS is observed: higher percentage of TDS (%TDS) corresponds to stronger links.

The TDS method (Bashan et al., 2012) to quantify the interaction between distinct physiologic systems A and B consists of the following steps (Figure 1). Consider the output signals {*a*} of system A and the output signal {*b*} of system B, each of length *N*. Divide both signals {*a*} and {*b*} into *N*_*L*_ overlapping segments ν of equal length *L* = 60*s*. Here we choose an overlap of *L*/2 = 30*s*, which corresponds to the time resolution of conventional sleep-stage-scoring epochs, and thus *N*_*L*_ = ⌊2*N*/*L*⌋−1, where ⌊2*N*/*L*⌋ is the largest integer *k* such that *k* ≤ 2*N*/*L*. Normalize the signals separately in each segment ν to zero mean and unit standard deviation in order to remove constant trends in the data and to obtain dimensionless signals. This normalization procedure assures that the estimated coupling between the signals {*a*} and {*b*} is not affected by their relative amplitudes. Then, calculate the cross-correlations

$$C_{ab}^{\nu }\left(\tau \right)=\frac{1}{L}\overset{L}{\underset{i=1}{\sum }}a_{i+\left(\nu -1\right)L/2}^{\nu }b_{i+\left(\nu -1\right)L/2+\tau }^{\nu }$$

between {*a*} and {*b*} in each segment ν using periodic boundary conditions. For each segment ν, we estimate the time delay $\tau_{0}^{\nu }$ as the maximum in the absolute value of the cross-correlation function $C_{ab}^{\nu }\left(\tau \right)$ in the segment (Figure 1).

These steps result in a new temporal series of time delays $\left\{\tau_{0}^{\nu }{|}_{\nu \in \left\{1,\ldots ,N_{L}\right\}}\right\}$ describing the temporal evolution of the cross-talk between the signals {*a*} and {*b*}. Time periods of stable interrelation between two signals are represented by segments of approximately constant τ_0_ in the series of time delays. In contrast, the absence of stable coupling between the signals corresponds to large fluctuations in τ_0_. To identify periods of stable coupling, the series of time delays is scanned using a 5 points sliding window (corresponding to a window of 5 × 30 s consecutive segments ν) with step size 1. Periods are labeled as stable when at least four out of five points the time delay remains in the interval [τ_0_ − 1, τ_0_ + 1] (Figure 1). The %TDS is finally calculated as the fraction of stable points in the time series $\left\{\tau_{0}^{\nu }\right\}$, and is a measure of the coupling strength between the two systems A and B.

### 2.4. Surrogate Tests and Significance Threshold for Network Links Strength

To test the statistical significance and physiological relevance of the network interactions identified by TDS method, we perform a surrogate test to establish a threshold of significance for links strength. Statistical significance is estimated by comparing the strength distribution of a given link obtained from all subjects in a given sleep stage with the distribution of the corresponding surrogate link representing “interactions” between the same two systems paired from different subjects.

A significance threshold for network links strength is determined performing the following steps: for each link in a given sleep stage, 200 surrogates are generated considering signals from two distinct and randomly chosen subjects, and a surrogate average link strength (%TDS) is obtained. The procedure is repeated for each network link to obtain a distribution of surrogate link strengths in each sleep stage. For each distribution the mean μ_*surr*_ and standard deviation σ_*surr*_ are estimated. Thus, the significance threshold at 95% confidence level for the network links strength is defined as μ_*surr*_ + 2σ_*surr*_ for each sleep stage. The significance threshold is represented by horizontal green lines in all figure panels showing bar plots of average links strength.

### 2.5. Cortico-Muscular Interaction Networks

#### 2.5.1. TDS Matrix and Network Link Definition

The TDS matrix consists of the pairwise coupling strength between seven cortical rhythms (δ, θ, α, σ, β, γ_1_, and γ_2_) derived from an EEG channel and each EMG frequency bands representing chin and leg muscle activation (Figure 2). The coupling strength between two signals is defined as the percentage of time over which TDS is observed, i.e., $\%TDS=\left(\overset{N_{L}}{\underset{i=1}{\sum }}s_{i}\right)/L\cdot 100$ where *s*_*i*_ is 1 if the corresponding *i*-th segment is labeled as stable for the TDS measure (red dots in Figure 1) or 0 if the corresponding *i*-th segment is labeled as unstable for the TDS measure (black dots in Figure 1) and *L* is the total duration of signals.

For each physiologic state, we calculate a group-average TDS matrix for couplings of each chin EMG (leg EMG) frequency band with each cortical rhythm from each of the EEG channels (Frontal Fp1 and Fp2; Central C3 and C4; Occipital O1 and O2). In these matrices each element represents the TDS coupling strength between signal *a* and *b* during a given sleep stage *s* averaged over all subjects and defined as:

(1) $$\begin{array}{l} {\bar{\%TDS}}_{ab}^{s}=\frac{\sum_{i=1}^{M}TDS_{i}\cdot L_{i}^{s}}{\sum_{i=1}^{M}L_{i}^{s}}\cdot 100, \end{array}$$

where $L_{i}^{s}$ represents the total duration of a given sleep stage *s* for subject *i*, and *TDS*_*i*_ stands for the TDS coupling strength between signal *a* and *b* for sleep stage *s* obtained from subject *i*.

To avoid artifacts related to the specific behavior of a subject (excessive movement, turning in bed, etc.) or to the specific record (electrode pops, poor electrode contact, salt bridge, etc.) affecting the estimation of coupling strength between two different signals during a given sleep stage, we remove the outliers according to the following procedure. We first calculate the mean *M* and standard deviation *SD* of the distribution of %TDS over all subjects for a given pair of signals during a given sleep stage, and then only subjects within the range [*M* − 2*SD, M* + 2*SD*] is included in the average procedure for this particular link.

**Figure 3:**
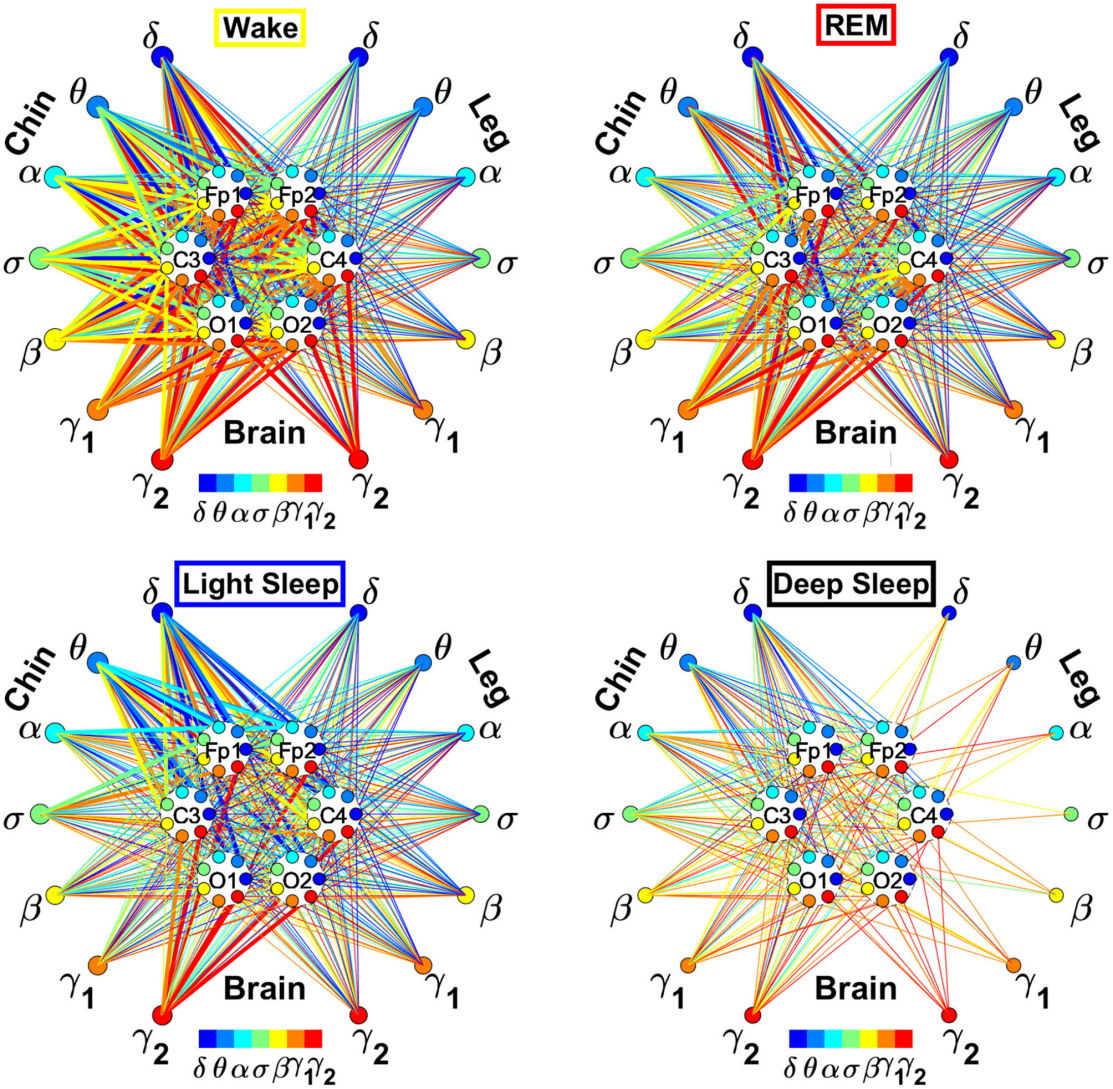
Dynamic networks of cortico-muscular interactions across physiological states. Network maps are obtained based on the group-averaged TDS matrices in Figure 2 representing physiological interactions during wake, REM, light, and deep sleep. Network links correspond to the TDS matrix elements, and show the coupling strength between seven physiologically relevant brain waves (δ, θ, α, σ, β, γ_1_, γ_2_) across cortical locations and muscle tone EMG frequency bands. Brain areas are represented by Frontal (Fp1 and Fp2), Central (C3 and C4), and Occipital (O1 and O2) EEG channels, where color nodes in each brain area represent distinct brain waves. Peripheral nodes indicate corresponding EMG frequency bands of chin and leg muscle tone shown in same color code as the brain waves. Links reflect the coupling strength between cortical rhythms at different locations and EMG frequency bands as quantified by the TDS measure (Materials and Methods 2.3). Links strength is marked by line width—thin lines for 3% < *%TDS* < 12%; thick lines for *%TDS* > 12%. All links above the threshold *%TDS* = 3% are shown; link color corresponds to the color of brain wave node involved in the interaction. A complex reorganization of network topology and links strength is observed with transition from one sleep stage to another, indicating a remarkable association between functional networks of cortico-muscular interaction and physiological states.

In the cortico-muscular network (Figure 3), brain areas are represented by Frontal (Fp1 and Fp2), Central (C3 and C4), and Occipital (O1 and O2) EEG channels, where nodes with different color in each brain area represent distinct brain waves. Peripheral nodes indicate EMG frequency bands of chin and leg muscle tone shown in the same color code as the brain waves. Network links show the coupling strength of each cortical rhythm across cortical areas with an EMG frequency band. Links strength corresponds to the matrix elements in Figure 2 and is marked by line width: thin lines for 3% < *%TDS* < 12%; thick lines for *%TDS* > 12%.

#### 2.5.2. Network of Interactions Between Cortical Rhythms and Integrated EMG Activity

To obtain information on the relative contribution of each brain rhythm on a given EEG channel with the integrated EMG activity, we consider the average coupling strength of a given brain wave from a given EEG channel with all EMG bands. We coarse-grain the matrices in Figure 2 by taking the average of the matrix elements along a given column, which means the average coupling of the integrated EMG activity with each cortical rhythm Δ*f*_*j*_, *j* = 1, ..., 7 from a cortical location; the average is given by

(2) $$\begin{array}{l} n_{1h}=\frac{1}{7}\overset{7}{\underset{EMG\left(\Delta f_{i}\right):i=1}{\sum }}\bar{\%TDS}\left[EMG\left(\Delta f_{i}\right),Brain\left(\Delta f_{j}\right)\right] \end{array}$$

where *h* = 7(*k* − 1) + *j*, *k* = 1, ..., 6 corresponding to a given EEG channel, and *%TDS*[*EMG*(Δ*f*_*i*_), *Brain*(Δ*f*_*j*_)] is the group-average %TDS between the frequency band Δ*f*_*i*_ of EMG and the cortical rhythm Δ*f*_*j*_ at a given EEG channel.

We develop a radar-chart representation to map such interactions from across different brain areas (**Figures 6**, **8**). This network consists of (i) six heptagons, one for each of the six brain areas corresponding to the locations of the EEG channels, and (ii) a centered hexagon representing the chin (**Figure 6**) or the leg (**Figure 8**). Nodes in the heptagons are color-coded according to the following scheme: dark blue for δ, light blue for θ, turquoise for α, green for σ, yellow for β, orange for γ_1_, and red for γ_2_. Brain heptagons are connected to the organ hexagon by links whose thicknesses encode the corresponding coupling strength. Networks include only links above a statistically significant threshold (section 2.4). The radar-chart centered in the organ hexagon represents the relative contribution to muscle control from different brain areas. The length of each segment along each radius in the radar-charts represents TDS coupling strength between each cortical rhythm at each EEG channel location and chin (**Figure 6**) or leg (**Figure 8**) muscle tone.

#### 2.5.3. Network of Interactions Between EMG Frequency Bands and Integrated EEG Activity

Similarly, in order to obtain information on the relative contribution of each EMG frequency band on a given EMG muscle tone with the integrated EEG activity, we consider the average coupling strength of a given EMG frequency band with all brain waves from a given EEG channel. We coarse-grain the matrices in Figure 2 by taking the average of the matrix elements along a given row, which means for each EMG frequency band Δ*f*_*i*_, *i* = 1, ..., 7 the average coupling strength with the *k*-th EEG channel is given by

(3) $$\begin{array}{l} m_{ik}=\frac{1}{7}\overset{7}{\underset{Brain\left(\Delta f_{j}\right):j=1}{\sum }}\bar{\%TDS}\left[EMG\left(\Delta f_{i}\right),Brain\left(\Delta f_{j}\right)\right]. \end{array}$$

This type of network represents the response of a EMG band to signals from the brain. The focus is to understand the role of each EMG band in the brain-muscle communication, for instance if there is preferential EMG frequency, and if there is physiologic state specific pattern in the cross-talk (**Figures 11**, **13**). Each network is constituted by six heptagons representing the six EEG channels, whose spatial distribution reminds the physical locations of electrodes on the brain surface from an axial point of view (Fp1, C3 and O1 on the left side and Fp2, C4, and O2 on the right side). Each of them represents the entire power spectrum of the corresponding EEG channel. The peripheral nodes represent the 7 frequency bands identified in the power spectrum of the chin (**Figure 11**) or leg (**Figure 13**) EMG muscle tone. The links between each node and a heptagon represent interactions of a given EMG band with each cortical location averaged over all cortical rhythms as defined in Equation (3); color of links and nodes corresponds to the frequency bands. Only the links with a TDS ≥ 3% are plotted; the thickness depends on the coupling strength. In particular, there are three different types of link thickness: thin links with 3% ≤ TDS < 5%, intermediate links with 5% ≤ TDS < 7.5% and thick links with TDS ≥ 7.5%.

### 2.6. Statistical Tests

The following statistical tests are used to validate the results: ANOVA test for group comparison and *t*-test for pair-wise comparison in case data passed the Kolmogorov-Smirnov normality test; otherwise, Kruskal-Wallis One Way Analysis of Variance on Ranks (ANOVA on Ranks) for group comparisons, and Mann-Whitney Rank Sum (MW) test for pair-wise comparisons. We perform Student-Newman-Keuls (SNK) Algorithm for multiple pairwise comparisons, since this method is robust against violations of normality. All statistical tests are performed on SigmaStat.

## 3. Results

### 3.1. Brain-Muscle Network and Its Dynamical Reorganization Across Physiologic States

We identify and characterize the brain-muscle interactions network across four major physiologic states: Wake, REM, LS and DS. We consider brain activity from six major cortical areas—frontal left-Fp1, frontal right-Fp2, central left-C3, central right-C4, occipital left-O1, and occipital right-O2, chin muscle tone, and leg muscle tone, simultaneously recorded over night-sleep using EEG and EMG (Materials and Methods section 2.1). To identify physiologic-state-specific communication pathways in the brain-muscle cross-talk, at each brain and peripheral muscle locations we decompose the recorded signals in seven physiologically relevant frequency bands—δ, θ, α, σ, β, γ_1_, and γ_2_. Thus, each location can be represented by seven network nodes, which may dynamically interact among them (intra-channel interactions) and with nodes in different locations (inter-channel interactions).

We then quantify pair-wise coupling and network interactions by means of the TDS method (Materials and Methods section 2.3). This novel approach is based on the concept of TDS (Bashan et al., 2012), and identifies periods of stable time delay between coordinated bursts in the output dynamics of diverse systems, as illustrated in Figure 1. Persistence of stable time delay between systems indicates stable interactions, and correspondingly stronger coupling between systems results in longer periods of TDS (Figure 1).

In Figure 2 we show the TDS matrices representing brain-chin and brain-leg interactions across physiologic states (Materials and Methods section 2.3). For each EEG channel, the matrix elements show the coupling strength between the seven physiological relevant cortical rhythms and the corresponding frequency bands of chin and leg EMG. We observe that both brain-chin and brain-leg TDS interaction matrices exhibit a clear stratification across sleep-stages: the coupling of cortical rhythms with EMG bands tends to be stronger during Wake and weaker during DS, and takes intermediate values during REM and LS (Figure 2). This observation demonstrates that, during Wake, bursts of cortical rhythms tend to be synchronized with a certain time delay with bursts of EMG activity, and the synchronization gradually decreases with transition to REM and LS, becoming minimal during DS. Indeed, the average number of synchronized bursts per minute—corresponding to periods of time delay stability—is 0.21 ± 0.08 for Wake, 0.17 ± 0.07 during REM, 0.15 ± 0.05 during LS, and 0.08 ± 0.04 during DS.

Importantly, the TDS matrices indicate that the contribution of specific cortical rhythms in brain-muscle communication depends on the particular physiologic state. During Wake, high frequency cortical rhythms, specifically γ_1_ and γ_2_, are the main mediators of the brain-chin and brain-leg interaction, strongly interacting with all EMG frequency bands (Figure 2). High frequency cortical rhythms play a dominant role also during REM, where they tend to be more strongly coupled to the corresponding high frequency bands of both chin and leg EMG.

In contrast to the high frequency cortical rhythms, we observe that slower cortical rhythms—i.e., δ, θ, α, and σ—become prominent in the brain-chin communication during light and deep sleep, and exhibit stronger interactions with the low frequency bands of the chin muscle tone (Figure 2). This pattern of interactions is not present in the brain-leg TDS matrices during light and deep sleep, where we find a predominance of γ_2_*EEG*__ − γ_2_*EMG*__ and γ_1_*EEG*__ − γ_1_*EMG*__ interactions. Such differences between brain-chin and brain-leg interaction patterns may relate to the differences between chin and leg muscle architecture, e.g., fiber types and fiber arrangement. The observed changes in the interaction pattern between brain waves and rhythms of muscle activation with the transition from one physiologic state to another reveal an intriguing dependence of cortico-muscular communications on physiologic states.

To better visualize and dissect the information provided by the TDS method, we next map the previously obtained TDS matrices into networks whose nodes and links represent the brain EEG and muscle EMG frequency bands and their pair-wise coupling (Figure 3). Nodes corresponding to EEG frequency bands in a specific scalp location form a heptagon. Six heptagons, each for one EEG channel, are located at the vertices of a hexagon representing the brain. Network nodes with different colors represent different cortical rhythms and EMG frequency bands. Network links show the interactions of cortical rhythms and EMG bands with thickness representing coupling strength and link color corresponding to the involved cortical rhythm (Figure 3).

We observe that the cortico-muscular network reorganizes across physiologic states. Specifically, the network is denser and exhibits stronger links during Wake, and tends to become sparser during LS and DS. Brain-chin network links are generally stronger than brain-leg links, in particular during Wake and REM (Figure 3). Importantly, we notice that cortico muscular links are strong also during REM, despite the muscle atonia that characterizes this physiologic state. This complex reorganization in the communication network is marked by the emergence of cortical rhythms and EMG frequency bands as main mediators of the brain-muscle interaction. During Wake and REM the strongest links correspond to interactions mediated by the high frequency cortical rhythms, and in particular γ_2_ (red links), indicating their prominent role in brain-muscle communication (Figure 3). We observe that the network markedly reorganizes with transition to LS, and strong links related to slower cortical rhythms appear in the network (dark and light blue links), in particular between the chin and the frontal region of the brain. Finally, the link number and strength abruptly decline during DS, revealing a marked difference between brain-chin and brain-leg communication (Figure 3).

### 3.2. Cortico-Muscular Interaction Profile of Network Links Strength as Hallmark of Sleep Stages

#### 3.2.1. Coarse-Grained Interaction Networks of Cortical Rhythms With Integrated Muscle Tone

**Figure 4:**
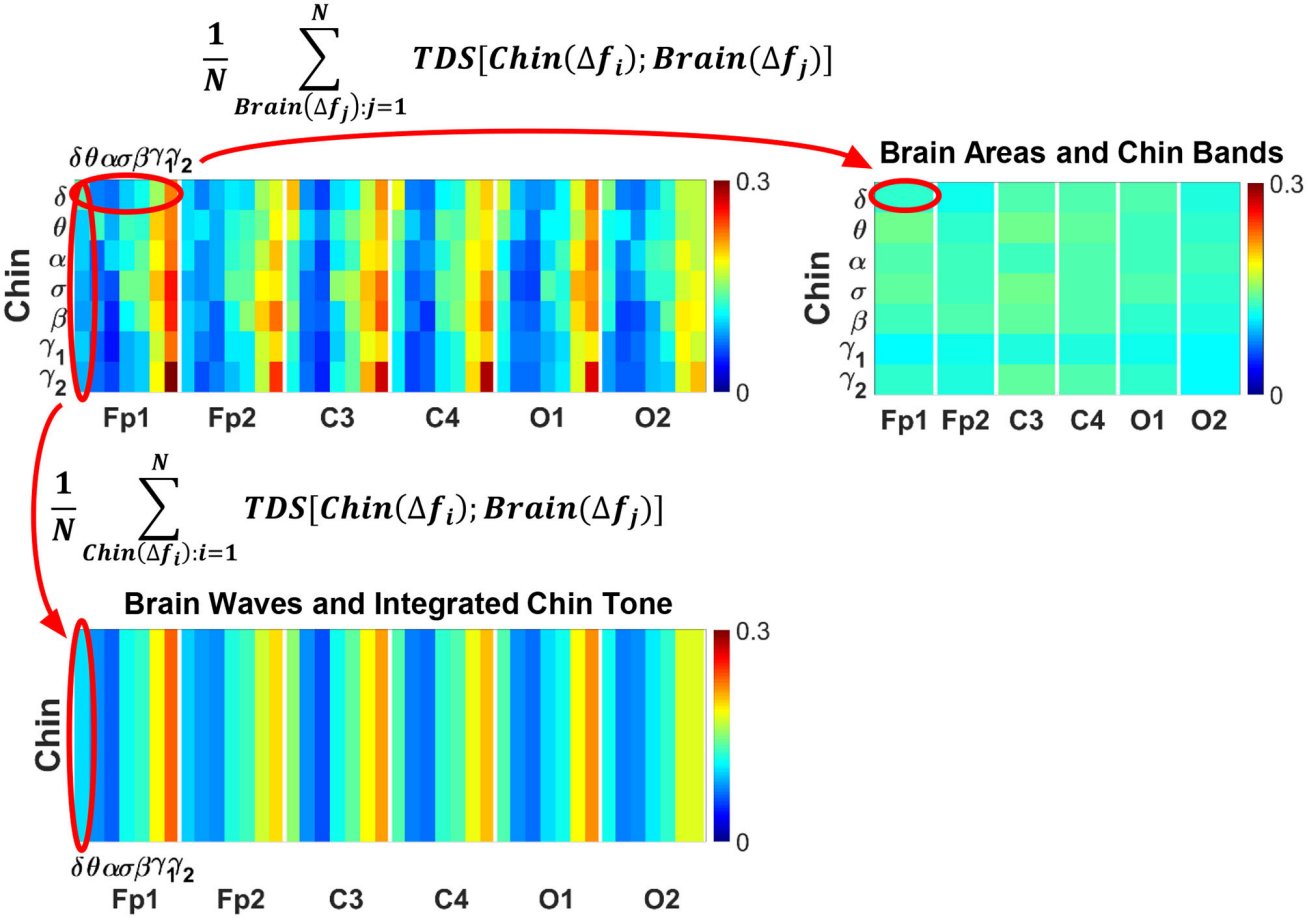
Schematic presentation of coarse-graining procedure for brain-muscle network interactions. (Top left panel) Group-averaged Time Delay Stability (TDS) matrix representing interactions between different brain waves (δ, θ, α, σ, β, γ_1_, γ_2_) and the corresponding chin muscle tone EMG bands during wake (as shown in Figure 2A). Coarse-graining the TDS matrix is essential to assess the relative contribution of each brain wave or EMG frequency band in the network of brain-muscle interactions. (Top right panel) Coarse-grained matrix of brain-muscle interaction where each matrix element (horizontal red oval) shows the average coupling strength of a given EMG band with all brain waves derived from a particular EEG channel location. Coarse-graining the TDS matrix across brain waves provides information on the relative contribution of each muscle EMG band in the communication with different brain areas. (Bottom panel) Coarse-grained matrix of brain-muscle interaction where each matrix element (vertical red oval) shows the average coupling strength of a given brain wave from a given EEG channel with all EMG bands. This coarse-graining of the TDS matrix across EMG frequency bands quantifies the contribution of different brain waves and brain locations to the brain-locomotor cross-talk, identifying the main mediators of the brain-to-muscle interaction.

To identify the role of different brain rhythms in muscle control across cortical locations, we coarse-grain the TDS matrix by taking an average across rows, i.e., EMG frequency bands, for each cortical rhythm column (Figure 4, bottom panel). Similarly, in order to investigate the relative contribution of each EMG bands in the brain-muscle interactions, we coarse grain the TDS matrix by averaging the elements along each brain wave row (Figure 4, right panel). These two average coarse-grained matrices are referred to as brain-to-muscle and muscle-to-brain interaction matrices.

**Figure 5:**
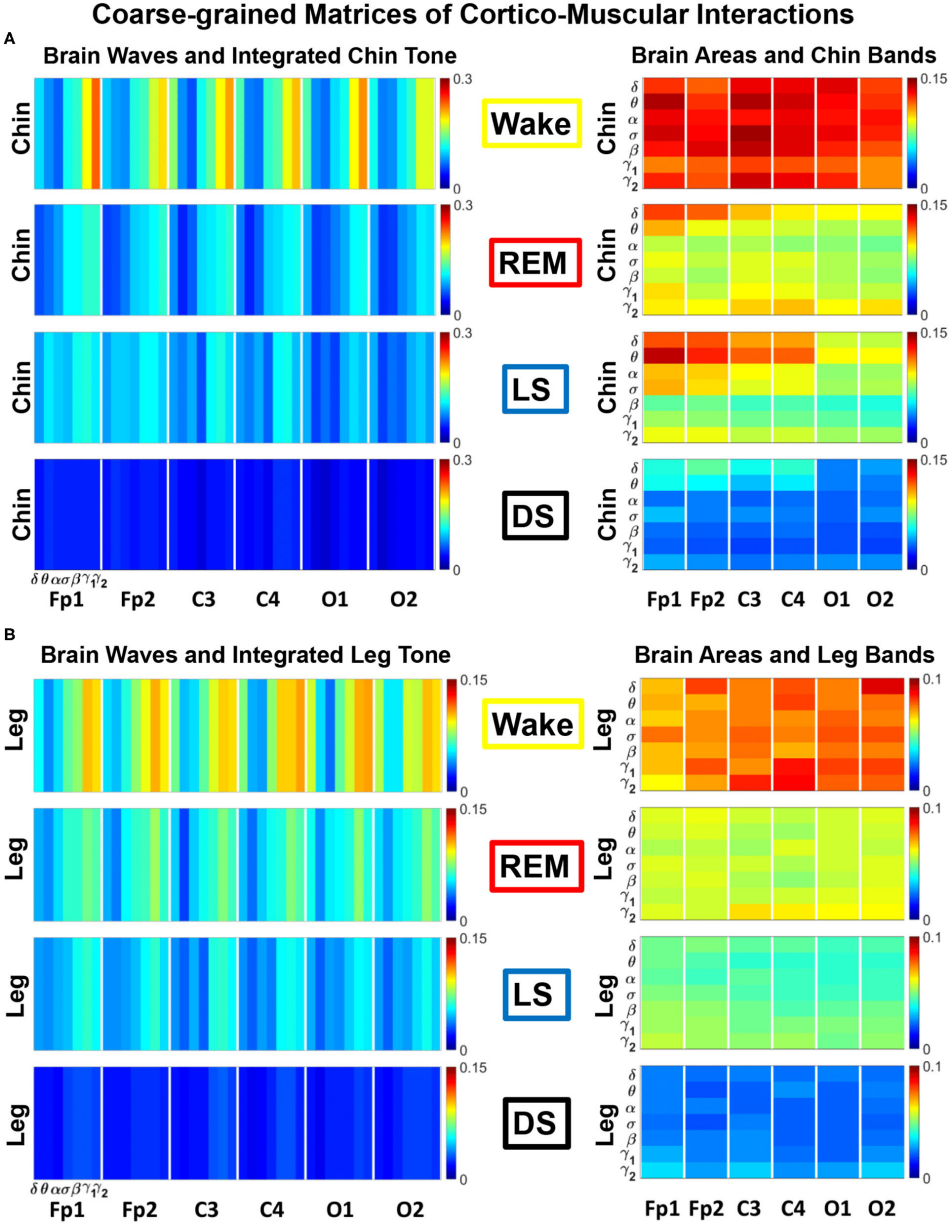
Dominant channels of communication and reorganization in cortico-muscular network interactions across physiological states. Group-averaged matrices of coupling strength (measured as *%TDS*; see Materials and Methods 2.3) for **(A)** brain vs. chin muscle tone and **(B)** brain vs. leg muscle tone interactions coarse-grained as shown in Figure 4 to represent the average coupling of (i) each brain rhythm at a given cortical location with integrated spectral power of all EMG frequency bands (left panels in **A,B**), and (ii) each individual EMG frequency band with integrated spectral power of all cortical rhythms for different brain locations (right panels in **A,B**). Both brain-chin and brain-leg networks exhibit pronounced reorganization with transition across sleep stages—strong coupling during wake, intermediate during REM and light sleep, and weak coupling during deep sleep—consistently present for both types of coarse-grained matrices (left vs. right panels in **A,B**). Notably, for each sleep stage, high frequency cortical rhythms exhibit stronger TDS coupling across all cortical areas (EEG channels), playing role as dominant channels and main mediators in both brain-to-chin and brain-to-leg networks interactions (marked by warm colors in left panels in **A,B**).

The results of such coarse-graining procedure are shown in Figure 5. The structure of the coarse-grained interaction matrices markedly changes across physiologic states, showing a reorganization in the communication pathways both in the brain-to-muscles (Figure 5, left panels) and muscle-to-brain networks (Figure 5, right panels). The brain-to-muscles interaction matrices clearly show the dominant role of high frequency cortical rhythms during Wake and REM, and a more relevant contribution of slower rhythms during LS in both chin and leg (Figure 5, left panels). Alternatively, the muscle-to-brain interaction matrices indicate that, while during Wake most frequency bands are significantly involved in the muscle-to-brain communication, low frequency EMG bands play an important role during REM, LS, and DS (Figure 5, right panels), in particular in the chin-to-brain communication over the frontal and central brain areas—Fp1, Fp2, C3, and C4 (Figure 5A, right panel).

Differently from chin, the leg-to-brain coarse-grained matrices exhibit a more uniform pattern of interactions across physiologic states (Figure 5B, right panel), and do not show predominance of low frequency EMG bands in the interaction with the brain during REM, LS, and DS.

On the one hand, changes in the mechanism of physiologic regulation impact the coordinated activation of different brain rhythms and their communication with myoelectrical activation (Figure 5, left panels). On the other hand, a particular mosaic of the profile of muscular rhythms interacting with a given brain location may uniquely define each physiologic state and different muscle groups (Figure 5, right panels).

##### 3.2.1.1. Dynamic Networks of Cortical Rhythms and Integrated Chin-muscle Tone

**Figure 6:**
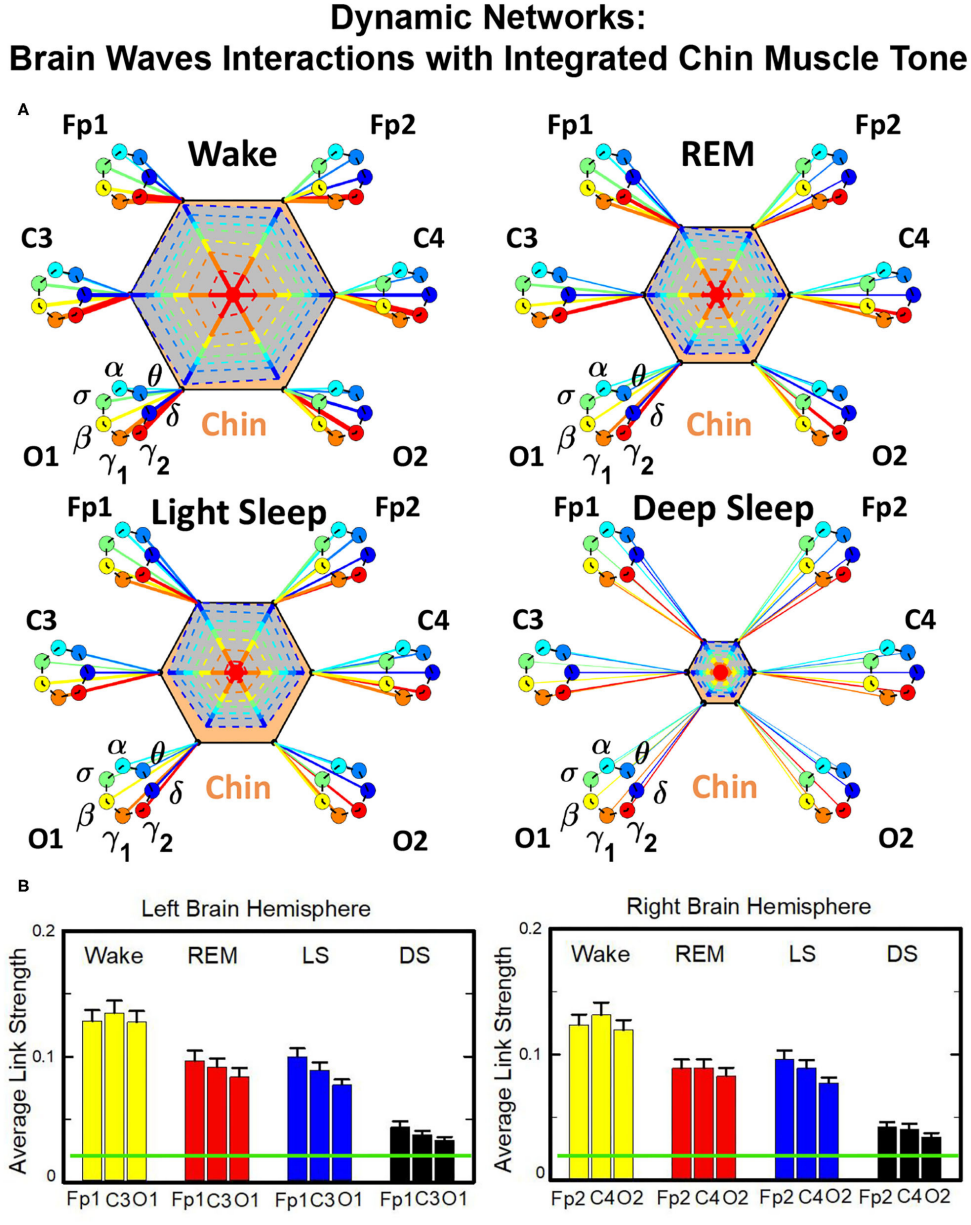
Dynamic networks of interaction between cortical rhythms and integrated chin-muscle tone across physiological states. **(A)** Links in network maps represent group-averaged TDS coupling strength (section Materials and Methods 2.5.1) between each brain rhythm at a given cortical location and the chin muscle tone, after averaging over all chin EMG bands (see Figure 4 and section Materials and Methods 2.5.2), and correspond to the elements in the coarse-grained matrices shown in Figure 5A, left panels. Brain areas are represented by Frontal (Fp1, Fp2), Central (C3, C4), and Occipital (O1, O2) EEG channels, and network nodes with different colors represent seven cortical rhythms (δ, θ, α, σ, β, γ_1_, γ_2_) derived from the spectral power of each EEG channel. Links strength is illustrated by line thickness, and links color corresponds to the color of brain rhythms (network nodes). Shown are all links with strength *%TDS* ≥ 2.3%, corresponding to the significance threshold based on surrogate tests (section Method 2.4). Radar-charts centered in the hexagons represent the relative contribution from different brain areas to the strength of network links during different sleep stages. The length of each segment along each radius in the radar-charts represents the TDS coupling strength between each cortical rhythm at each EEG location and chin muscle tone. The segments are shown with the same color as the corresponding brain rhythms (network nodes). The brain-chin network interactions are mainly mediated through high frequency γ_1_ and γ_2_ cortical rhythms (thicker orange and red links), and are characterized with relatively symmetric links strength to all six cortical areas, as shown by the symmetric radar-chart in each hexagon, with stronger contribution from the Frontal and Central areas. Network reorganization is observed with transition across sleep stages: with overall stronger network links during wake (larger hexagon), intermediate during REM and light sleep, and much weaker interactions (smaller hexagon) during deep sleep. **(B)** Histograms of links strength in the brain-chin network during different sleep stages. Group-averaged links strength is obtained using the TDS measure, where each bar represents the average strength of interaction of all cortical rhythms from a given brain area (Frontal, Central or Occipital) with all chin muscle tone EMG bands. Error bars represent the standard error obtained for all subjects in the group; horizontal green lines in both panels mark a surrogate test threshold (*%TDS* = 2.3%; section Method 2.4) above which network interactions are physiologically significant. A pronounced sleep-stage stratification pattern is observed for the average links strength related to each cortical area, consistent for both left and right hemisphere (pair-wise comparison between sleep stages for the same brain area gives *p* ≤ 0.05 (MW test), except for REM vs. light sleep, and one-way ANOVA rank test comparison across all sleep stages gives *p* ≤ 0.001). Brain-chin muscle tone network interactions exhibit strong symmetry in links strength between the left and right hemisphere for all sleep stages (MW test, *p* ≥ 0.65).

The brain-to-chin networks derived from the coarse-grained TDS matrices are shown in Figure 6. The brain-to-chin interaction network significantly changes with transition across sleep, with strong interactions during Wake, intermediate during REM and LS, and weak during DS (Figure 6). Our analysis of the brain-to-chin interaction network shows symmetric interaction of chin with right and left brain hemisphere for all sleep stages. Furthermore, the average link strength across different brain areas exhibits a non-uniform pattern, with a prevalence in strength for the links between chin and frontal areas (Fp1 and Fp2), as indicated by the radar chart inside the chin hexagon in Figure 6. A One-Way ANOVA rank test for the average link strength over brain locations (frontal, central and occipital) (Figure 5B) shows a statistically significant difference between sleep stages, with *p* ≤ 0.001 for both hemispheres (pairwise multiple comparison test: *p* < 0.05 for all pairs but REM vs. LS in both hemispheres; similar results are obtained for multiple and pairwise comparison at each brain location). No significant differences are found between hemispheres in each sleep stage.

To validate the results and the relation to underlying physiology, we performed additional tests. To confirm the physiological origin of cortico-muscular interaction pattern, we perform a surrogate test (Materials and Methods section 2.4), and obtain a significant threshold for coupling strength shown by green lines in each figure. All results presented in all bar plots show that the coupling strengths are above the physiological significance. Remarkably, the entire ensemble of cortico-muscular interaction profile is consistent when comparing all subjects in our database during the same physiologic state, indicating a universal mechanism underlying cortico-muscular interactions (error bars in Figure 6). These observations reveal that, at short time scales, there is a previously unrecognized complex organization of cortical and muscular rhythms interactions, which continuously coordinate during a given sleep stage and reorganize with transition across sleep stages.

**Figure 7:**
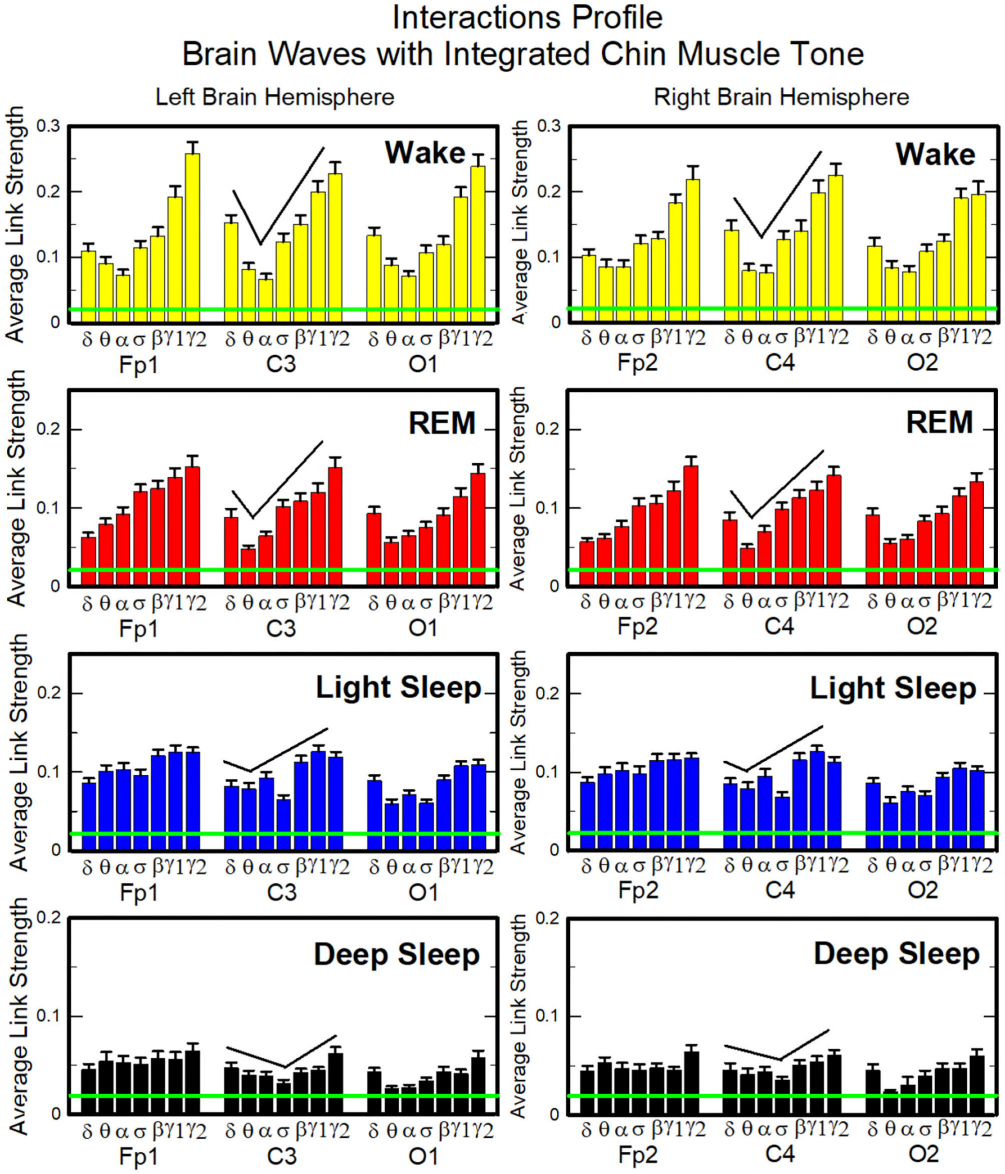
Characteristic profiles of network links strength for cortical rhythms interactions with integrated chin-muscle tone. Group-averaged links strength is obtained using the TDS measure, where each link represents the interaction of chin muscle tone (averaged over all EMG bands, as shown in Figures 4, 5A, left panel) with each cortical rhythm at a given brain area. Link strengths are grouped by brain areas (Frontal Fp1 and Fp2, Central C3 and C4, Occipital O1 and O2), and are ordered from low- to high-frequency cortical rhythms for each area, matching the network graph presentation of links between network nodes (cortical rhythms) in each brain location and the radar-charts (sum of interactions with all chin EMG bands) as shown in Figure 6A. A characteristic profile of network links strength as function of cortical rhythms frequency is consistently observed for all brain areas in both left and right hemisphere—strongest network interactions mediated through the high-frequency γ_2_ cortical rhythm, a gradual decrease in links strength for the lower-frequency rhythms (γ_1_, β, σ, α, θ), followed by increase in links strength for the lowest-frequency δ brain wave. This characteristic profile is well-pronounced during wake, REM and light sleep (one-way ANOVA tests *p* ≤ 0.001), and gradually flattens for deep sleep (one-way ANOVA test *p* = 0.3). Notably, the profile is robust, exhibiting almost identical shape and matching strength of network links within the profile for both left and right brain hemisphere during each sleep stage. The pronounced sleep-stage stratification observed for the network links strength in the brain-chin radar-charts (shown as change in size of hexagons in Figure 6A) is consistently present for the links to all cortical rhythms and brain areas—stronger links during wake, intermediate during REM and light sleep, and weaker links during deep sleep. Error bars represent the standard error obtained for all subjects in the group; horizontal green lines in both panels mark a surrogate test threshold (*%TDS* = 2.3%; section Method 2.4) above which network interactions are physiologically significant.

Next, we study the characteristic profile of network links strength (Figure 7). We find that for a given physiologic state, the frequency profile of brain-to-chin network links remains stable for all brain areas (Frontal, Central, and Occipital). However, comparing different physiologic states, we observe significant differences in the characteristic frequency profiles for the strength of brain-to-chin interactions. Specifically, during Wake frequency profiles are characterized by strongest links for the high-frequency bands γ_1_ and γ_2_ and a gradual decrease in links strength for the lower-frequency bands, followed by a slight kink up in link strength for the δ band (One-Way ANOVA rank test on Fp1: *p* < 0.001; pairwise multiple comparison test: *p* < 0.05). With transition to REM and LS, the frequency profiles remain mostly stable for all brain areas, with only the links strength between different frequency bands reduced compared to Wake. This is particularly evident in the C3 and C4 channels, which are closer to the motor cortex (Figure 7). The observed differences among links are still significant both in REM and LS (One-Way ANOVA rank test on Fp1: *p* < 0.001; pairwise multiple comparison test for δ, θ, and α links: *p* < 0.05; pairwise multiple comparison test between δ, θ, α links and the subset {σ, β, γ_1_, γ_2_}: *p* < 0.05).

During DS brain-to-chin interactions become weaker and the relative strength distribution is more homogeneous (Figure 7), although the high EEG frequency links remain stronger (One-Way ANOVA rank test on Fp1: *p* < 0.001; pairwise multiple comparison *p* = 0.310).

These distinct types of cortico-muscular networks indicate that interactions between different brain waves and integrated myoelectrical activity play different roles in physiologic regulation.

##### 3.2.1.2. Dynamic Networks of Cortical Rhythms and Integrated Leg-muscle Tone

**Figure 8:**
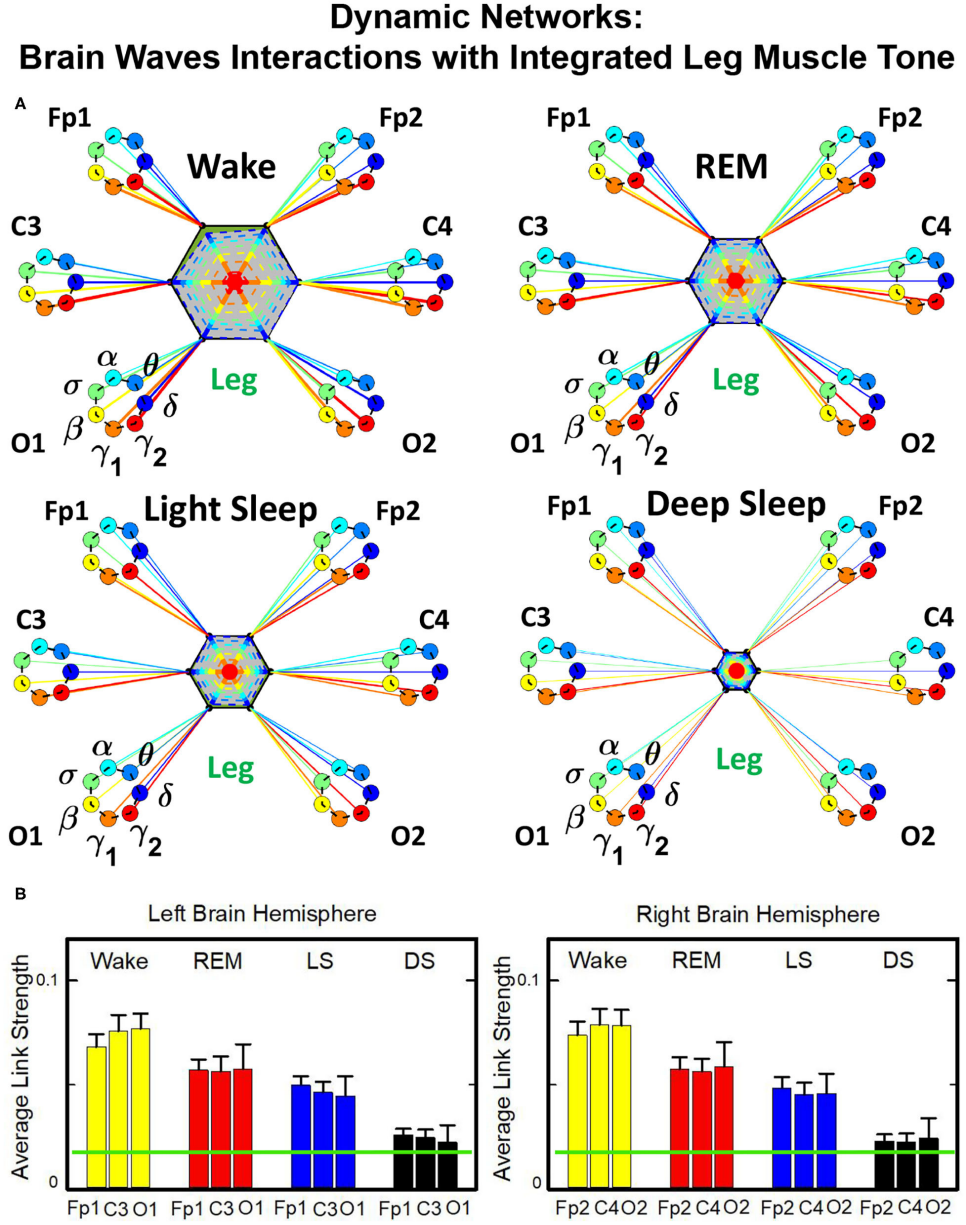
Dynamic networks of cortical rhythms and integrated leg-muscle tone interactions across physiological states. **(A)** Links in network maps represent group-averaged TDS coupling strength (section Materials and Methods 2.5.1) between each brain rhythm at a given cortical location and the leg muscle tone, after averaging over all leg EMG frequency bands (see Figure 4 and section Materials and Methods 2.5.2). Links correspond to the elements in the coarse-grained matrices shown in figure 5B left panels. Brain areas are represented by Frontal (Fp1, Fp2), Central (C3, C4), and Occipital (O1, O2) EEG channels, and network nodes with different colors mark cortical rhythms (δ, θ, α, σ, β, γ_1_, γ_2_). Links strength is indicated by line thickness; links color corresponds to the color of cortical rhythms (network nodes). Shown are links with strength *%TDS* ≥ 2.3%, corresponding to the significance threshold derived from surrogate tests (section Method 2.4). Radar-charts centered in the leg hexagons represent the relative contribution of different brain areas to the strength of network links during different sleep stages. The length of each segment along each radius in the radar-charts represents TDS coupling strength between each cortical rhythm at each EEG location and the leg muscle tone averaged over all EMG bands. Segments in the radar-charts are shown with same color as the corresponding brain rhythms (network nodes). Brain-leg network interactions are mainly mediated through high frequency γ_1_ and γ_2_ cortical rhythms (thicker orange and red links), and exhibit relatively symmetric links strength to all six cortical areas, as shown by the symmetric radar-chart in each hexagon. Networks reorganize with transition across sleep stages: stronger network links during wake (larger hexagon), intermediate during REM and light sleep, and much weaker interactions (smaller hexagon) during deep sleep. The sleep-stage reorganization in brain-leg network interactions is consistent with the brain-chin network (Figure 6). **(B)** Histograms of links strength in the brain-leg network during different sleep stages. Group-averaged links strength is obtained using the TDS measure, where each bar represents the average strength of interaction of all cortical rhythms from a given brain area (Frontal, Central or Occipital) with all muscle tone EMG bands. Error bars represent the standard error obtained for all subjects in the group; horizontal green lines in both panels mark a surrogate test threshold (*%TDS* = 2.3%; section Method 2.4) above which network links are significant. A pronounced sleep-stage stratification pattern is observed for the average links strength related to each cortical area, consistent for both left and right hemisphere (pair-wise comparison between sleep stages for the same brain area gives MW test *p* ≤ 0.05, and one-way ANOVA rank test comparison across all sleep stages gives *p* ≤ 0.001). Brain-leg network interactions exhibit strong symmetry in links strength between the left and right hemisphere for all sleep stages (MW test *p* ≥ 0.67).

Structure and evolution of the brain-to-leg network across sleep stages closely resemble the brain-to-chin networks (Figure 8). The interaction network significantly changes with transition across sleep stages, with stronger links during Wake, intermediate during REM and LS, and weak during DS (Figure 8). A One Way ANOVA rank test for the average link strength over brain locations (frontal, central, and occipital) shows a statistically significant difference between sleep stages, with *p* ≤ 0.001 for both hemispheres. The average link strength is symmetric between left and right hemispheres, and exhibits a uniform distribution across different brain areas, as indicated by the radar chart inside the leg hexagon in Figure 8 (One-Way ANOVA rank test gives *p* ≥ 0.438). No significant differences are found between hemispheres in each sleep stage (MW test, *p* ≥ 0.67).

**Figure 9:**
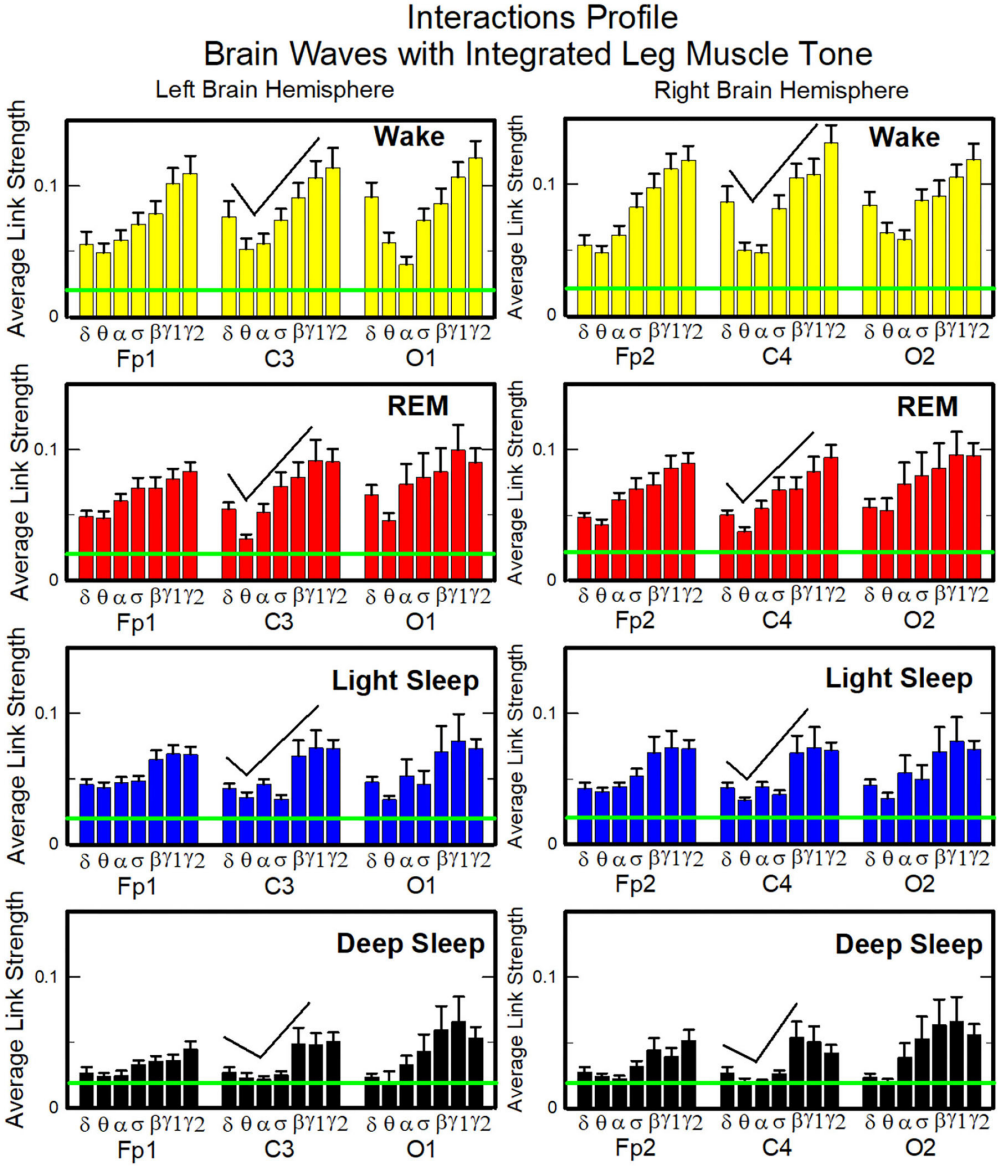
Characteristic profiles of network links strength for cortical rhythms interactions with integrated leg-muscle tone. Group-averaged links strength is obtained using the TDS measure, where each link represents the interaction of the leg muscle tone (averaged over all EMG bands, as in Figures 4, 5B, right panel) with each cortical rhythm at a given brain area. Link strengths are grouped by brain areas (Frontal Fp1 and Fp2, Central C3 and C4, Occipital O1 and O2), and are ordered from low- to high-frequency cortical rhythms for each area. Groups of bar charts represent network links between nodes (cortical rhythms) in each brain location and the radar-charts (sum of interactions with all chin EMG bands) as shown in Figure 8A. A consistent profile of links strength as function of cortical rhythms frequency is observed for all brain areas—strongest interactions mediated through the high-frequency γ_2_ cortical rhythm, a gradual decrease in links strength for the lower-frequency rhythms (γ_1_, β, σ, α, θ), followed by slight increase in links strength for the lowest-frequency δ brain wave. This characteristic profile is well-pronounced during all sleep stages (one-way ANOVA tests *p* ≤ 0.002, indicating statistical significance when comparing all links in the profile). The sleep-stage stratification pattern observed for network links in the brain-leg radar-charts (change in size of hexagons in Figure 8) is also consistently present for all links mediated by cortical rhythms across brain areas—stronger links during wake, intermediate during REM and light sleep, and weaker links during deep sleep. A strong symmetry in the shape of network links profiles and links strength is observed between left and right hemisphere for each sleep stage. Remarkably, both brain-leg (panels above) and brain-chin (Figure 7) networks exhibit similar links strength profile for muscle tone and cortical rhythms interactions, indicating universal network dynamics and mechanism of regulation. Error bars represent the standard error obtained for all subjects in the group; horizontal green lines in both panels mark a surrogate test threshold (*%TDS* = 2.3%; Section Method 2.4) above which network interactions are physiologically significant.

The analysis of the brain-to-leg network shows that the frequency profile of network links remains stable for all brain areas (Frontal, Central and Occipital) in a given sleep stage. During Wake, brain-to-leg interaction is characterized by strongest links for the high-frequency bands γ_1_ and γ_2_ and a gradual decrease in links strength for the lower-frequency bands, followed by a slight kink up in link strength for the δ band (One-Way ANOVA rank test on Fp1: *p* < 0.002; pairwise comparison between θ and all other frequency bands: *p* < 0.05; group comparison between the subsets {δ, α, σ, β}, and {γ_1_, γ_2_}: *p* < 0.05). A similar frequency profile characterizes the network of interactions both during REM, LS, and DS, and differences across frequency bands remain significant (One-Way ANOVA rank test on Fp1: REM, *p* < 0.002; LS and DS *p* < 0.001; REM pairwise comparison: δ, θ, and γ_2_ are different from each other—SNK test *p* < 0.05—, and they are significantly different from the subset {α, σ, β, γ_1_}). Importantly, high-frequency cortical rhythms dominate brain-to-leg communication in all sleep stages, in particular γ_2_, whose link strength is significantly higher also during DS (SNK test *p* < 0.05) (Figure 9).

Our findings demonstrate the need to extend the traditional framework of understanding physiologic states through the prism of interactions of cortical rhythms with muscle activation at large time scales. In addition to this classical picture, we find that for a given physiologic state, there is a unique interaction network structure of cortico-muscular communications. Further, the same sleep-stage stratification pattern in the strength of cortico-muscular network interactions is consistently observed for each individual subject as well as for the group average, indicating a universal mechanism underlying communications among brain waves and muscular rhythms.

##### 3.2.1.3. Interaction Between Cortical Rhythms and Muscle Tone Frequency Bands

Our analysis of coarse-grained TDS matrices shows the role played by different brain rhythms in muscle control across physiologic states (Figures 5–9). Next, we analyze the fine structure of the brain-muscles interaction network and ask how different cortical rhythms interact with muscle activity in specific frequency bands.

**Figure 10:**
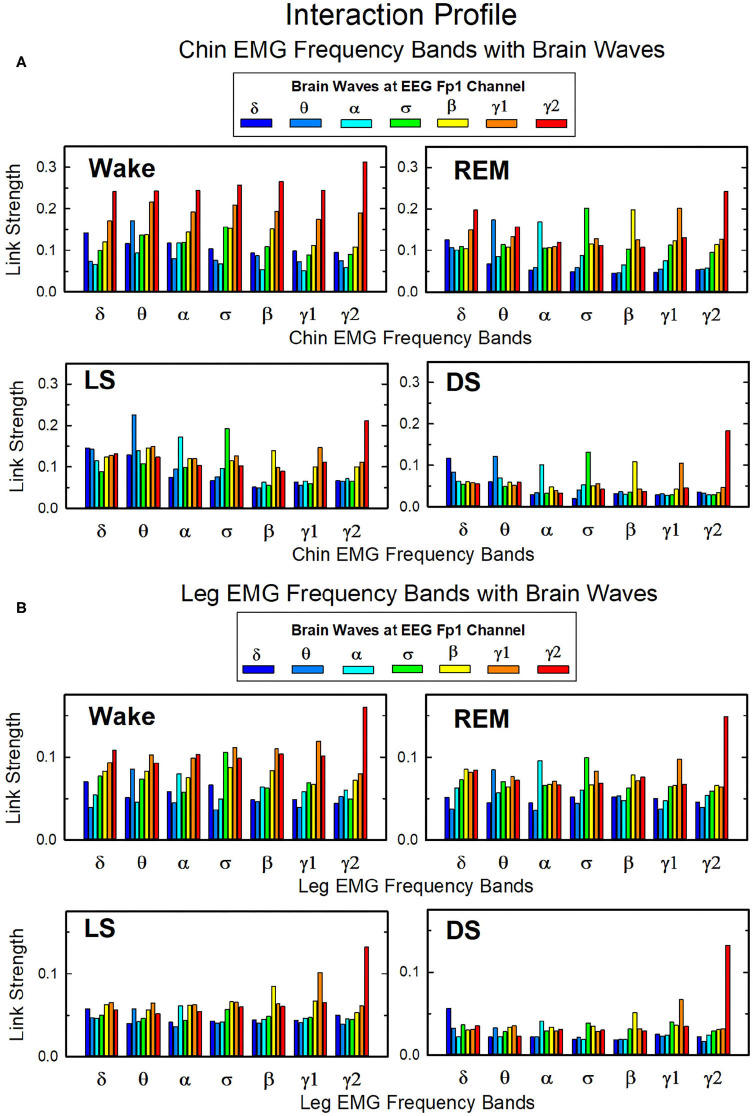
Network links of interaction between cortical rhythms and muscle tone frequency bands. Histograms of group-averaged of network links as measured by the TDS method (Materials and Methods section 2.3) representing the strength of interaction between brain cortical rhythms (δ, θ, α, σ, β, γ_1_, γ_2_) and the corresponding EMG frequency bands of **(A)** chin and **(B)** leg muscle tone for different sleep stages. Shown are network links for cortical rhythms from the Frontal Fp1 area. Links are grouped to present the interaction of each cortical rhythm with all EMG bands. Figure panels show the Fp1 profile of network links strength (Figures 7, 9) for different sleep stages—the average links strength of each group in a panel is presented as a separate bar in the Fp1 profile in Figures 7, 9. Histograms show an inhomogeneous distribution of links strength, where interactions tend to be stronger for links between same frequency cortical rhythms and EMG frequency bands—e.g., δ_*EEG*_-δ_*EMG*_, θ_*EEG*_-θ_*EMG*_, etc., where the γ2_*EEG*_-γ2_*EMG*_ coupling is particularly pronounced. This tendency is more evident in the brain-chin compared to the brain-leg network, and during REM, light and deep sleep compared to wake (pair-wise comparison between links in each group gives *p* < 0.05 (MW test) for the γ2_*EEG*_-γ2_*EMG*_, indicating statistical significance of network links mediated by same EEG and EMG frequency bands).

In Figure 10 we show the strength of interactions between cortical rhythms and the corresponding EMG frequency bands of chin and leg muscle tone during each sleep stage. We observe that cortical rhythms do not interact with muscles uniformly through all EMG frequency bands. For instance, during Wake the γ_2_ EEG rhythm preferentially interacts with the γ_2_ EMG band, the θ EEG rhythm with the θ EMG band, and the δ EEG rhythm with the δ band of chin and leg EMG. During REM, LS, and DS, we find that each cortical rhythm tends to interact more strongly with the EMG activity in the same frequency band, particularly in the communication with the chin (Figure 10A).

Overall, we observe that the γ_2_*EEG*__ − γ_2_*EMG*__ interaction tends to be the strongest channel for the brain-muscle communication in all sleep-stages, for both chin and leg. However, the contribution of different cortical rhythms in brain-muscle communication depends on the particular physiologic state. During Wake, high frequency cortical rhythms generally show the strongest interactions with all EMG frequency bands (Figure 10). High frequency cortical rhythms play a dominant role also during REM, where they exhibit a stronger coupling with the corresponding high frequency bands of both chin and leg EMG.

On the other hand, during REM, LS, and DS, we observe that slower cortical rhythms—i.e., δ, θ, α, and σ—tend to have a similar contribution as γ_1_ and γ_2_, and same-frequency interactions become prominent.

#### 3.2.2. Coarse-Grained Interaction Networks of Integrated Brain Activity at Cortical Locations and Muscle Activation Frequency Bands

##### 3.2.2.1. Network Interactions of Cortical Areas With Chin-EMG Frequency Bands

**Figure 11:**
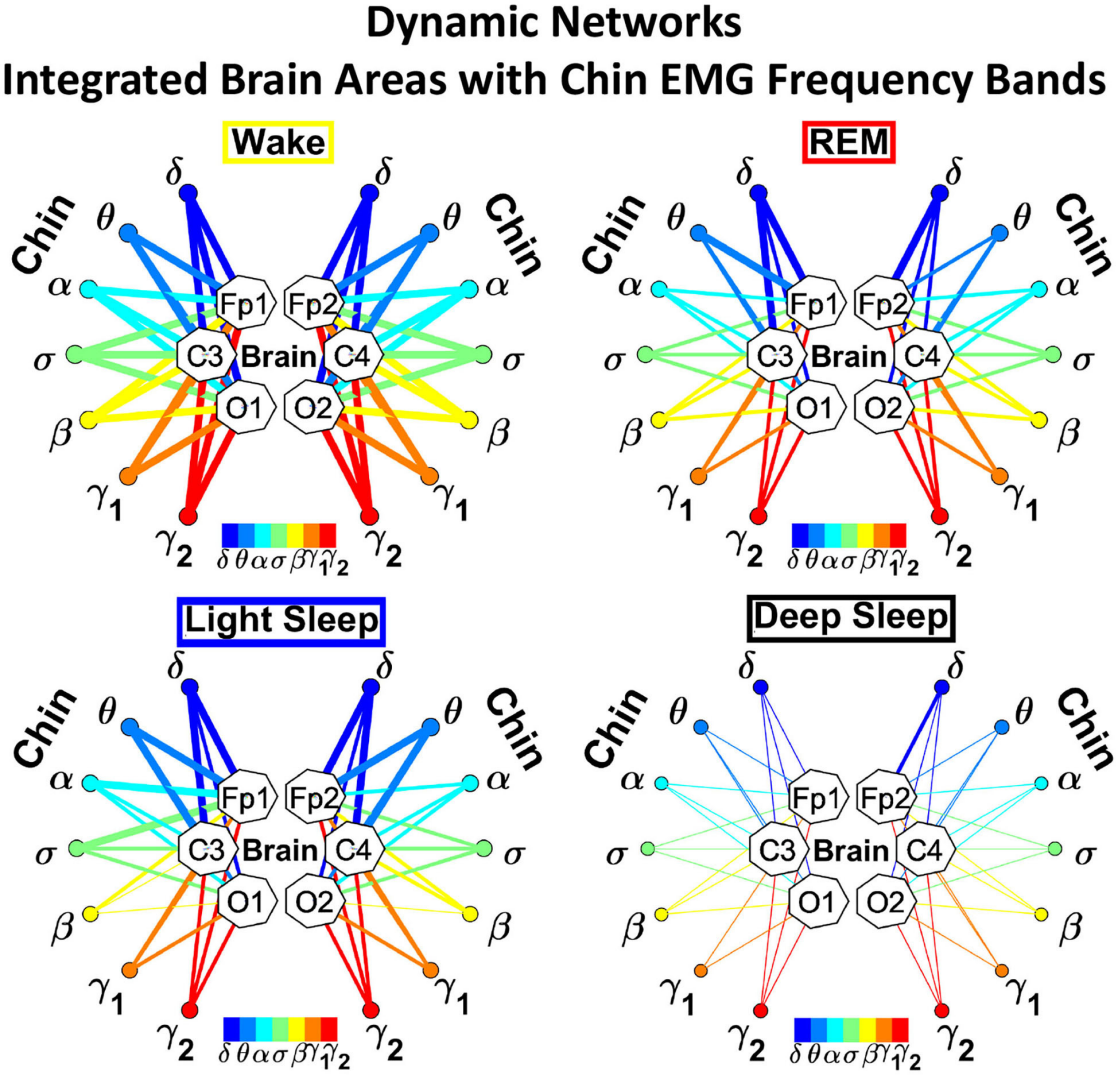
Dynamic networks of individual chin EMG frequency bands and integrated brain dynamics at cortical locations for different physiological states. Links in network maps represent group-averaged TDS coupling strength (section Materials and Methods 2.5.1) between each frequency band of chin muscle tone and a given cortical location, after averaging over all brain waves (see Figure 4 and section Materials and Methods 2.5.3), and correspond to the elements in the coarse-grained matrices shown in Figure 5A, right panels. Brain areas are represented by Frontal (Fp1, Fp2), Central (C3, C4), and Occipital (O1, O2) EEG channels, while peripheral network nodes with different colors represent the chin muscle tone frequency bands. Line thickness indicates link strength (thin links with 3% ≤ TDS < 5%, intermediate links with 5% ≤ TDS < 7.5% and thick links with TDS ≥ 7.5%), and links color corresponds to the color of the EMG network nodes. The chin-to-brain communication network and its dominant pathways depend on the physiologic state (sleep stage). All links across EMG bands are strong during wake independently of the brain area. During REM and LS we observe stronger links between low-frequency chin EMG δ and θ bands and the frontal (Fp1 and Fp2) and central (C3 and C4) brain areas. Links are generally weaker during DS, and the strongest links are those connecting δ and θ bands to the frontal areas.

**Figure 12:**
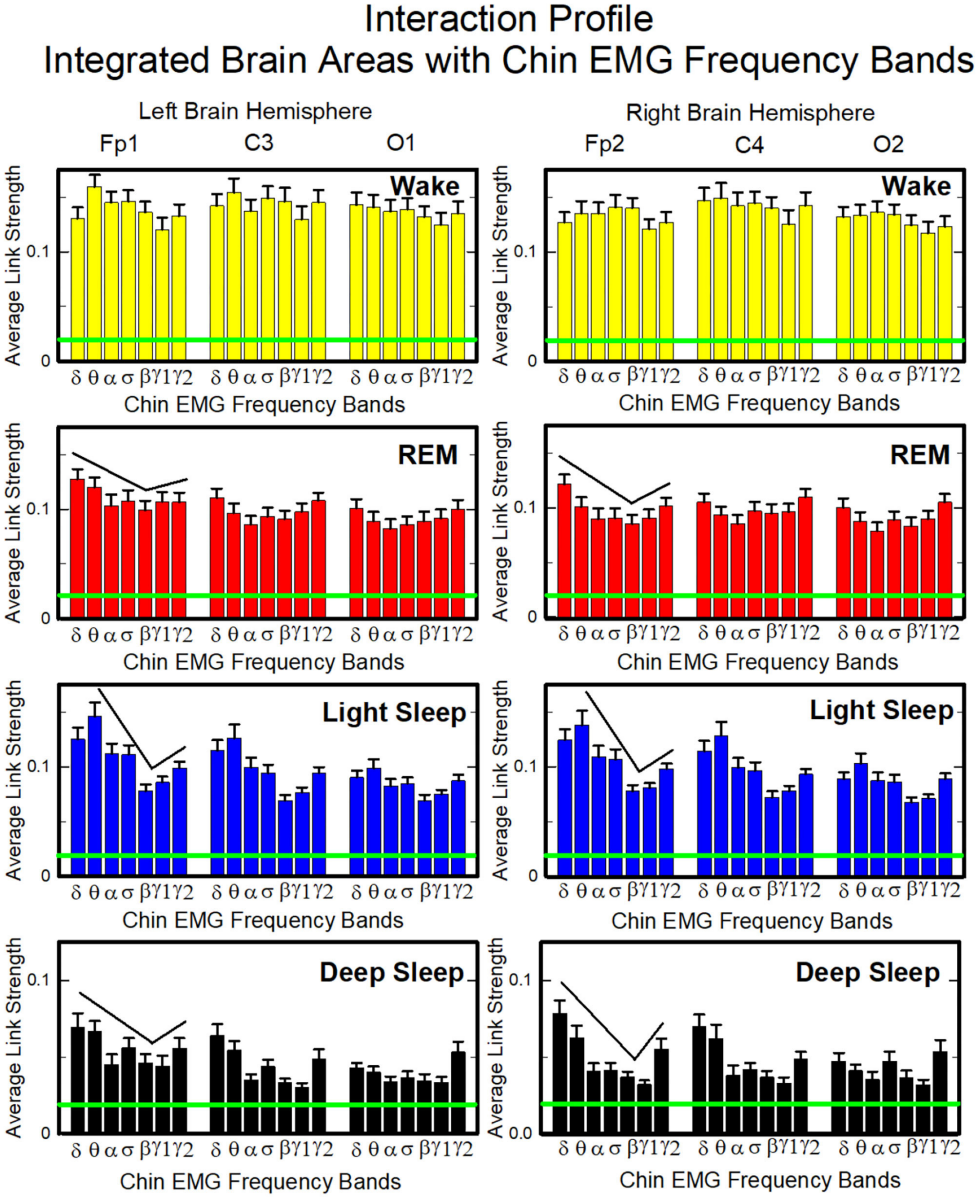
Characteristic profiles of network links strength representing interactions between integrated brain activity at cortical areas and individual chin-EMG frequency bands. Group-averaged links strength is obtained using the TDS method (Materials and Methods section 2.3), where each link represents the interaction of brain activity from a given cortical area (averaged over all brain waves derived from the EEG channel located at this cortical area) and each muscle tone rhythm (frequency band) derived from the chin EMG signal. Links are grouped by brain cortical areas in both left and right hemisphere (Frontal Fp1 and Fp2, Central C3 and C4, Occipital O1 and O2; marked on top of the panels), and are ordered from low- to high-frequency chin EMG bands. Bars indicate the strength of links shown on the network maps in Figure 11. The displayed profiles provide detailed information on the interaction between averaged cortical activity at a given EEG channel location with each individual chin EMG frequency band. Error bars represent the standard error; horizontal green lines mark a threshold *%TDS* = 2.3% based on a surrogate test (section Method 2.4) above which network interactions are physiologically significant with >97% confidence level. A characteristic profile of links strength is associated with each physiological state (sleep stage)—uniform distribution of links strength across EMG bands for all cortical brain areas during wake, and dominance of low-frequency chin EMG δ and θ bands during REM, light and deep sleep (pair-wise MW tests comparing links mediated by δ and θ EMG bands vs. any other links between EMG bands and a given cortical area show statistically significant difference with *p* ≤ 0.05). Links strength profiles show clear symmetry between left and right hemisphere (pair-wise MW tests *p* ≥ 0.65) with a gradual decline in links strength from the Frontal to Central and Occipital areas.

To investigate the relative contribution of each muscle EMG band in the communication with different brain areas, we next consider the average coupling strength of a given EMG frequency band with all brain waves derived from a particular EEG channel (Figure 4, right panel). Similar to the brain-chin interaction network, chin-brain communication network also reorganizes across physiologic states (Figure 11). Comparing profiles of cortico-muscular interactions for different physiologic states, we discover that each state is characterized by a specific ensemble of profiles, universal for all subjects (error bars in Figure 12). During Wake, the distribution of links strength across EMG frequency bands for all cortical areas is rather uniform, with a corresponding nearly flat frequency profile (Figure 12) (One-Way ANOVA rank on the Fp1 bars group: *p* ≥ 0.211). On the other hand, we observe that the links corresponding to low-frequency δ and θ EMG bands tend to be dominant during REM, light and deep sleep (thicker dark and light blue links) (Figure 11). A One-Way ANOVA rank test shows that differences in link strengths are significant (*p* ≤ 0.002). In particular, pairwise comparison indicates that the interactions between chin θ band and Fp1 are significantly stronger than the interactions of Fp1 with all the other chin frequency bands during LS (SNK test, *p* < 0.05). Likewise, during DS interactions between δ and θ bands and Fp1 are significantly stronger than the interactions between Fp1 and all the other chin frequency bands (SNK-test, *p* < 0.05). Similar results are found on the central areas of the brain during REM, LS and DS in both hemispheres (Figure 12). Importantly, link strengths are symmetric between left and right hemispheres, with a dominant contribution in the frontal areas of both hemispheres.

##### 3.2.2.2. Network Interactions of Cortical Areas With Leg-EMG Frequency Bands

**Figure 13:**
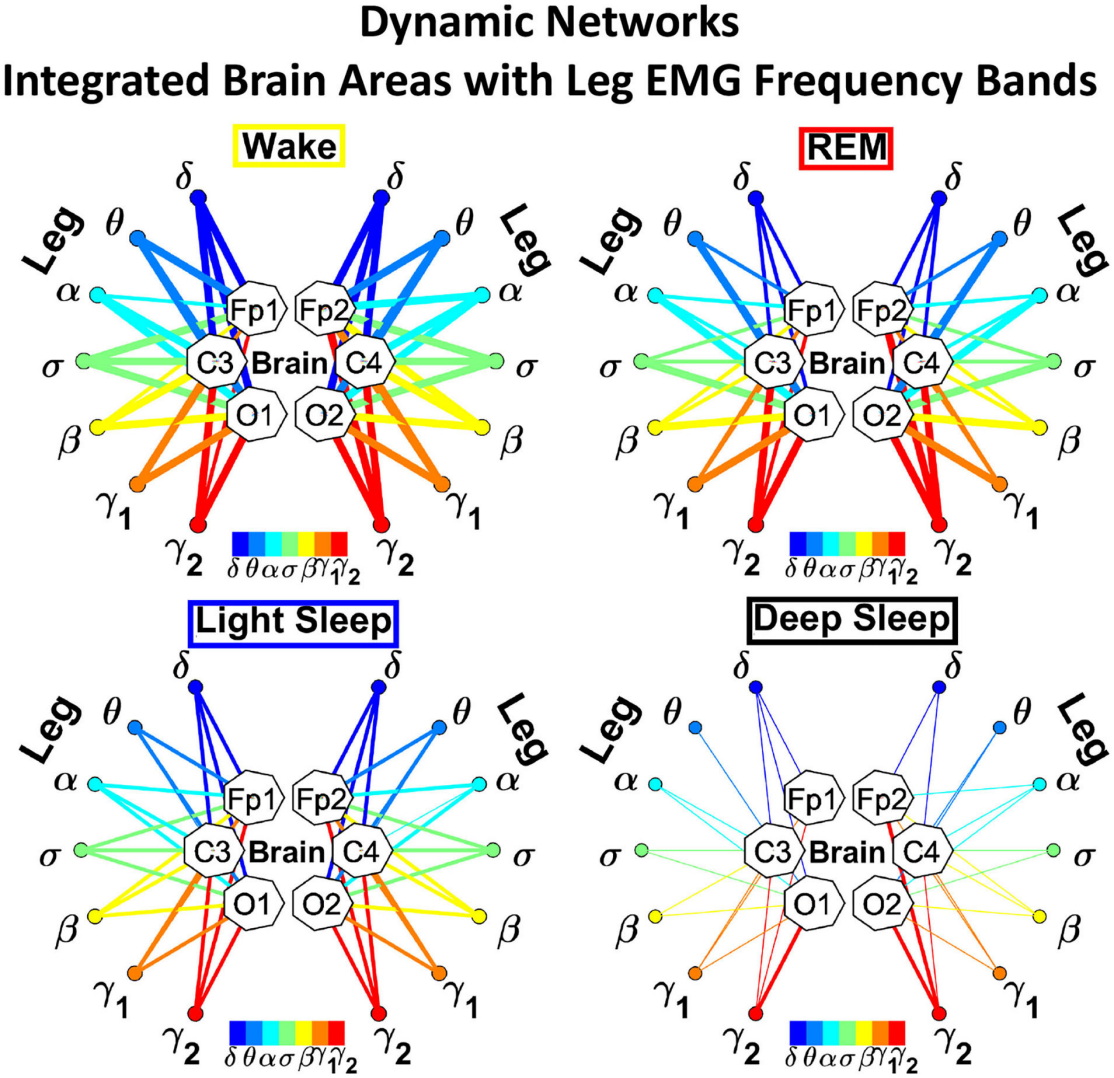
Dynamic networks of individual leg EMG frequency bands and integrated brain dynamics at cortical locations for different physiological states. Links in network maps represent group-averaged TDS coupling strength (section Materials and Methods 2.5.1) between each frequency band of leg muscle tone and a given cortical location, after averaging over all brain waves (see Figure 4 and section Materials and Methods 2.5.3), and correspond to the elements in the coarse-grained matrices shown in Figure 5B, right panels. Brain areas are represented by Frontal (Fp1, Fp2), Central (C3, C4), and Occipital (O1, O2) EEG channels, while peripheral network nodes with different colors represent leg EMG frequency bands. Line thickness indicates link strength (thin links with 3% ≤ TDS < 5%, intermediate links with 5% ≤ TDS < 7.5% and thick links with TDS ≥ 7.5%) and links color corresponds to the color of leg EMG frequency bands (network nodes). Network links are generally stronger during wake, and their strength uniformly declines with transition to REM, LS, and DS, which exhibits weak interactions across all frequency bands. No clear dominant communication pathways are observed in any of the four physiologic states.

**Figure 14:**
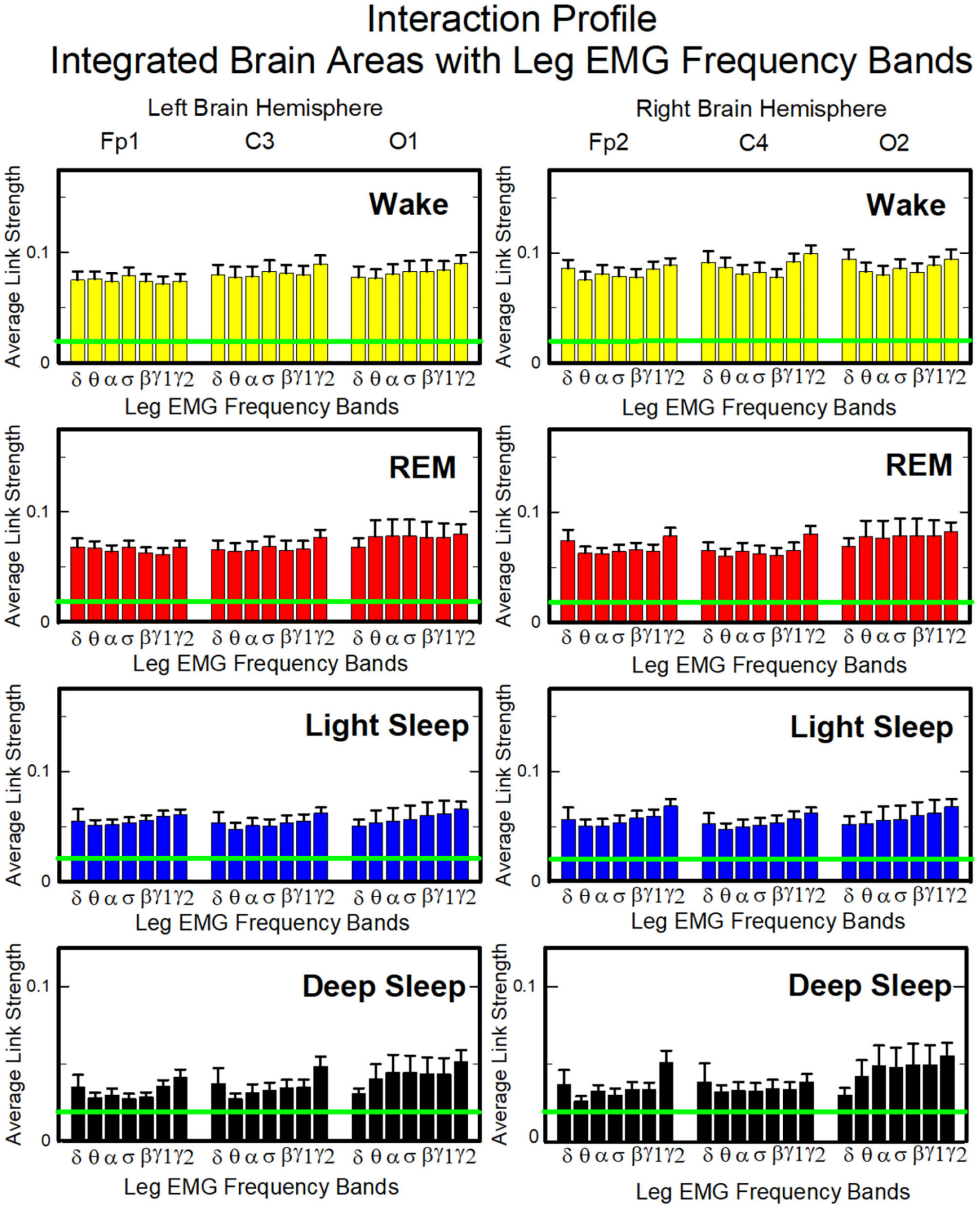
Characteristic profiles of network links strength representing interactions between integrated brain activity at cortical areas and individual leg-EMG frequency bands. Group-averaged links strength is obtained using the TDS method (Materials and Methods section 2.3), where each link represents the interaction of brain activity from a given cortical area (averaged over all brain waves derived from the EEG channel located at this cortical area) and each muscle tone rhythm (frequency band) derived from the leg-EMG signal. Links are grouped by brain cortical areas in both left and right hemisphere (Frontal Fp1 and Fp2, Central C3 and C4, Occipital O1 and O2; marked on top of the panels), and are ordered from low- to high-frequency leg-EMG bands. Bars indicate the strength of links shown on the network maps in Figure 13. Note, that the average strength of each group of links in the panels corresponds to a separate bar in Figure 8B, and the displayed profiles provide detailed information on the interaction between averaged cortical activity at a given EEG channel location with each individual leg-EMG frequency band. Error bars represent the standard error obtained for all subjects in the group; horizontal green lines mark a threshold *%TDS* = 2.3% (based on surrogate test, section Method 2.4) above which network interactions are physiologically significant with >97% confidence level. Bar-charts show absence of dominant links in the interactions of leg-EMG bands with cortical areas, and a flat profile of links strength across EMG bands for all cortical brain areas during all sleep stages (one-way ANOVA *p* ≥ 0.21, indicating no significant difference between links in each profile). Links strength profiles show clear symmetry between left and right hemisphere (pair-wise MW tests *p* ≥ 0.67, indicating no significant difference).

Our analysis of the leg-to-brain interaction network shows a rather uniform distribution of links over cortical areas, and a clear symmetry between left and right hemispheres (Figure 13). Differently from the chin-to-brain network, we do not observe dominant links in the interactions of leg EMG bands with cortical areas, and a flat profile of link strengths across EMG bands characterizes all cortical areas during all sleep stages (One-way ANOVA *p* ≥ 0.21). Links are generally stronger during Wake, and their strength gradually decreases from Wake to REM, LS and DS (Figure 14). Notably, these interaction profiles result from short scale synchronous modulation in brain waves and EMG amplitudes. The observed profiles of brain waves and EMG interactions indicate a hierarchical reorganization of the entire brain-muscle communication network with transition across physiologic states.

##### 3.2.2.3. Interaction Between Muscle Tone Frequency Bands and Cortical Rhythms

**Figure 15:**
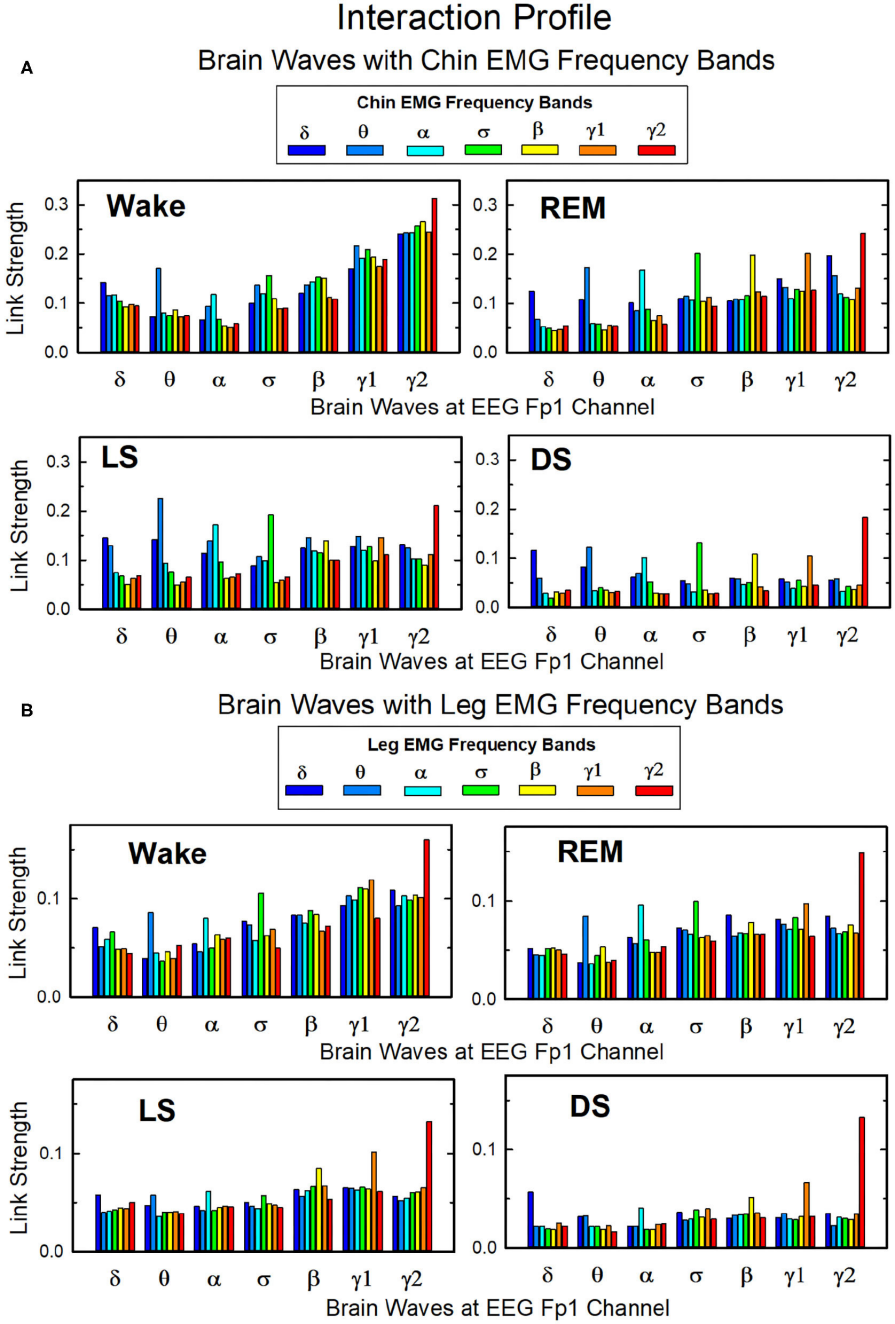
Network links of interaction between muscle tone frequency bands and cortical rhythms. Histograms of group-averaged network links as measured by the TDS method (Materials and Methods section 2.3) representing strength of interaction between each EMG frequency band of **(A)** chin and **(B)** leg muscle tone and brain cortical rhythms (δ, θ, α, σ, β, γ_1_, γ_2_ from the Frontal Fp1 area) for different sleep stages. Links are grouped to present the interaction of each EMG band with all cortical rhythms at the Fp1 channel location. Each figure panel corresponds to the links strength interaction profile of all EMG bands with the Fp1 cortical area (shown in Figures 12, 14) for a given sleep stage—the average links strength of each group in a panel is presented as a separate bar in the Fp1 profile in Figures 12, 14. Histograms show inhomogeneous distribution of links strength, where all EMG bands of both chin and leg muscle tone exhibit dominant interactions with the high-frequency γ_1_ and γ_2_ cortical rhythms during wake (one-way ANOVA for each separate group gives *p* ≤ 0.001), while REM, light and deep sleep are characterized by stronger same-frequency coupling of EMG bands with the corresponding cortical rhythms (δ_*EEG*_-δ_*EMG*_, θ_*EEG*_-θ_*EMG*_, etc.), a behavior more pronounced for chin-brain compared to leg-brain interactions (Method 2.6). Results are consistent for all brain areas (EEG channel locations), indicating universal patterns in cortical rhythm and muscle tone network interactions.

Next, we analyze the fine structure of the muscle-brain interaction network and ask how muscle activity in specific EMG frequency bands interacts with different cortical rhythms. In Figure 15 we show the strength of the interactions between EMG frequency bands of chin/leg muscle tone and cortical rhythms during each sleep stage. We observe that all EMG frequency bands preferentially interact with the γ_2_ cortical rhythm during Wake, especially in the chin (Figure 15A). During REM, LS, and DS, each cortical rhythm tends to interact more strongly with the EMG in the same frequency band. This interaction pattern is more pronounced in the chin-to-brain communication. These findings indicate that muscular rhythms coordinate their activation in response to changes in physiologic regulation during different sleep stages, dynamically interacting with different cortical rhythms.

## 4. Discussion

We present a systematic empirical study of the brain-muscles interaction networks during the four major physiologic states—Wake, LS, REM, and DS. Unlike previous studies focusing on CMC under particular conditions (Conway et al., 1995; Boonstra et al., 2009; Cheyne, 2013), e.g., muscular contraction, here we investigate the synchronous activation between cortical rhythms and peripheral muscle activity at the integrated cortical level during sleep, and map the default brain-muscle network across physiologic states. We consider chin and leg muscle tone, and identify basic functional pathways of communication characterizing each physiologic state with no external perturbation and no conscious movement initiation.

We note that according to empirical findings reported in the literature, during REM sleep we have muscle atonia. Nevertheless, we need to carefully re-examine the concept of muscle atonia, that is usually referred to muscle EMG activity with a very small amplitude. Indeed, in the data we show that the amplitude of EMG muscle activity during REM, even during LS, is very low compared to wake (Figure 1). However, what our method identifies and quantifies is synchronous modulations in the EEG and EMG signals that are independent of the amplitude of the EMG signal. For instance, two signals with relatively small amplitude can have synchronous modulations and synchronous bursts, and thus relatively strong coupling, while two signals with large amplitude may have no synchronous modulations and no synchronous bursts, and, as a result, weak coupling. In other words, the concept of having high activity reflected in the large amplitude of the EMG is different from the concept of presence of coupling and interactions between two systems, which is independent of the size of the amplitude of their output signals. These are two very different concepts, and even in situations where we have signals with small amplitude, or one dominant signal with a large amplitude and another one with very small amplitude, they still can be coupled because of the presence of synchronous modulations (bursts) in their respective dynamics, where synchronous modulation indicates presence of coupling in our TDS method. Our results show that the cortico-muscular coupling is stronger during wake, weaker during REM and LS, and weakest during DS. This finding indicates that in REM both the amplitude of the EMG and the cortico-muscular coupling are lower compared to wake. However, we note that the cortico-muscular coupling during DS is weaker compared to LS, although the amplitude of the EMG does not significantly change with transition from LS to DS (Figure 1A). Thus, our findings provide new insights on muscle activity and its coupling with cortical rhythms across different physiologic states, which complements the current knowledge of physiologic regulation impacting the amplitude of EMG signals.

As previous findings show that physiological couplings between systems change with transitions from one physiologic state to another (Bartsch et al., 2012; Bartsch and Ivanov, 2014), we also find that the cortico-muscular interaction network shows a complex structure that reorganizes with transitions from one physiologic state to another (Figures 3, 6, 8, 11, 13), and can be described by unique cortico-muscular interaction profiles (Figures 7, 9, 12, 14). Our analysis shows that during wake the cortico-muscular network exhibits high connectivity, and the coupling between cortical rhythms and EMG frequency bands is stronger. Network connectivity and link strength gradually decrease with transitions to REM and LS, and further during DS, where we observe very sparse networks of weak links (Figure 3).

Furthermore, we demonstrate the existence of preferred pathways of communication between brain and peripheral muscles that uniquely characterize the brain-muscle interaction network across physiologic state. Specifically, we find that contribution of cortical rhythms to brain-muscles communication depends on the physiologic state, and that cortical rhythms preferentially couple with specific EMG frequency bands. The reported results show that: (i) γ_1_ and γ_2_ rhythms play a prominent role in the communication with both chin and leg, particularly during wake and REM; (ii) slower rhythms—δ, θ, α, σ, and β—become strongly involved in the interaction between brain and muscles during REM, LS, and DS, and predominantly couple with the corresponding frequency bands of chin/leg. Remarkably, we observe that cortico-muscular links are rather strong also during REM (Figures 7, 9, 12, 14), indicating a considerable level of cortico-muscular synchronization despite the muscle atonia typical of REM sleep (Krenzer et al., 2011). In particular, we find that the brain-muscles interactions are stronger during REM than during DS, although muscles are more active during DS, a previously unrecognized characteristic in the autonomic regulation of skeletal muscles.

Overall, we observe that the interaction γ_2_*EEG*__ − γ_2_*EMG*__ tends to be the strongest channel for the brain-muscle communication in all sleep-stages, in both chin and leg (Figures 10, 15). Coupling between high-frequency cortical rhythms and high EMG frequency bands for both muscles is particularly strong in the C3 and C4 EEG channels across all sleep stages. This is due to the proximity of C3 and C4 to the primary sensorimotor cortex and the primary motor cortex, located immediately posterior and anterior to the central sulcus, respectively (Fox et al., 2001; Mayka et al., 2006).

Importantly, we find that cortical rhythms and EMG frequency bands involved in brain-muscle communication, as well as the strength of their mutual interaction, may also depend on the specific muscle fibers and on their structural arrangement. In particular, we show that the role of slow cortical rhythms is more pronounced in the brain-chin interaction network, a fact that may be related to chin muscle architecture and functions. Indeed, 84% of the chin muscle fibers are hybrid fibers, an unusual combination of fibers type I and II identified only in cranial muscles and responsible of unique functions like chewing, swallowing, respiration, and movements that require precise control over muscle activity (Takahashi et al., 2002). During sleep respiratory rate goes down and the suprahyoid muscles of the chin, which have the role of keeping the airway opened to facilitate breathing, work at low frequencies. Correspondingly, during LS and DS, we observe prominent interactions between slow cortical rhythms and equivalent frequency bands of EMG chin muscle tone—strong TDS coupling δ_*EEG*_-δ_*EMG*_, θ_*EEG*_-θ_*EMG*_, and α_*EEG*_-α_*EMG*_ (Figures 10, 15). On the other hand, leg muscles do not play an active role during sleep, and the brain-leg interactions through low EMG frequency bands are weaker than interactions involving high EMG frequency bands. Moreover, we also observe that brain-leg interactions involving high EMG frequency bands are weaker than brain-chin interactions. This may relate to the fiber composition of the tibialis anterioris, which mostly consists of slow fibers (about 80%) (Jaworowski et al., 2002) contracting at low frequencies. Comprehensively, brain-leg interactions are generally weaker than brain-chin interactions, and this could be due to the non-primary role of leg muscles during sleep, while the submental muscle is involved in some crucial functions like jaw opening and respiration (Mu et al., 2004).

Our analysis shows that the default brain-muscle network comprises state-specific patterns of communication involving several frequency bands—not only β or γ as shown by CMC during motor contraction (Conway et al., 1995; Brown et al., 1998; Baker et al., 1999; Omlor et al., 2007). Our network approach provides a first demonstration of how brain rhythms coordinate collectively as a network to control muscle activation during different physiologic states. Muscle fibers activation is maintained even at resting conditions and in the absence of directed movements. Our findings of statistically significant difference in the group average of network links strength across different sleep stages indicate change in the mechanism through which the brain regulates muscle activation in different sleep stages, and thus demonstrate a physiologically relevant change that is associated with a given physiologic state. We note that we perform two types of statistical test: (i) a statistical test comparing the strength of brain-muscle network interactions across physiological states where we find statically significant difference, and (ii) a surrogate test in order to determine the level of link strength beyond which a given link strength is not a result of random factors. In the latter we investigate the spurious coupling between signals which are actually not coupled to each other, since coming from different subjects. Therefore, our indication based on surrogate tests shows that we can't distinguish whether a link with a %TDS below 2.3% is physiologically relevant or not, but every link with strength in TDS measure above 2.3% has physiological meaning because indicates stronger coupling than one would observe by random chance between two uncoupled signals coming from two different subjects.

Importantly, we identify the main cortical rhythms and EMG frequency bands through which the default brain-muscle communication occurs during each physiologic state, and demonstrate universal laws in brain control of locomotor system. Indeed, reported results are robust and consistent across subjects. Studying the interaction between brain and muscles during sleep—when the muscle tone is low and is not related to specific physical activity—we are able to uncover physiologic mechanisms of autonomic regulation that do not depend on active locomotion but are function of the physiologic state. The results reported here demonstrate a strong association between the network of coordinated cortico-muscular communications and physiologic states. The distinct profiles of brain waves and muscle interactions across sleep stages redefine sleep through a previously unrecognized hierarchical network organization of cortical rhythms interactions, and open new perspectives on the regulatory mechanisms of brain dynamics and locomotor activation during sleep, with implications for novel biomarkers of sleep and movement disorders.

## Data Availability Statement

The data we used in this work are pre-existing multi-channel physiologic recordings from EU SIESTA databases. All participants provided written informed consent. The detailed protocol of the SIESTA database can be found in Klösch et al. (2001), http://ieeexplore.ieee.org/stamp/stamp.jsp?arnumber=932725. Requests to access these datasets should be directed to http://www.ofai.at/siesta/.

## Ethics Statement

All participants provided written informed consent. Data use and protocol were approved by the Boston University Charles River Campus Institutional Review Board (IRB protocol number 3380X) and were conducted according to the principles expressed in the Declaration of Helsinki.

## Author Contributions

PI conceived the idea and designed the study. XZ organized the database. RR and FL performed data pre-processing. RR, XZ, JW, and PI performed the analyses and prepared the figures. RR, XZ, JW, FL, and PI performed the research, interpreted the results, and designed the results visualization. RR, FL, and PI wrote the manuscript. All authors contributed to manuscript revision, read, and approved the submitted version.

## Conflict of Interest

The authors declare that the research was conducted in the absence of any commercial or financial relationships that could be construed as a potential conflict of interest.
